# Small-molecule flunarizine increases SMN protein in nuclear Cajal bodies and motor function in a mouse model of spinal muscular atrophy

**DOI:** 10.1038/s41598-018-20219-1

**Published:** 2018-02-01

**Authors:** Delphine Sapaly, Matthieu Dos Santos, Perrine Delers, Olivier Biondi, Gwendoline Quérol, Léo Houdebine, Kevinee Khoobarry, François Girardet, Philippe Burlet, Anne-Sophie Armand, Christophe Chanoine, Jean-François Bureau, Frédéric Charbonnier, Suzie Lefebvre

**Affiliations:** 1 Université Paris Descartes, Sorbonne Paris Cité, UFR des Sciences Fondamentales et Biomédicales, INSERM UMR 1124, Paris, F-75006 France; 2 Laboratoire de Biologie Cellulaire des Membranes, Institut Jacques-Monod, Centre National de la Recherche Scientifique (CNRS) UMR 7592, Université Paris Diderot, Sorbonne Paris Cite, France; 3 INSERM UMR 393, Université Paris Descartes, Paris, France; 4 Unité de la Génétique Fonctionnelle des Maladies Infectieuses, Institut Pasteur, Paris, France; 5 CNRS URA3012, F-75015 Paris, France; 6 Present Address: INSERM U1016, Institut Cochin, Université Paris Descartes, Paris, France; 7 Present Address: INSERM U1151, Institut Necker Enfants Malades (INEM), Université Paris Descartes, Paris, France

## Abstract

The hereditary neurodegenerative disorder spinal muscular atrophy (SMA) is characterized by the loss of spinal cord motor neurons and skeletal muscle atrophy. SMA is caused by mutations of the survival motor neuron (SMN) gene leading to a decrease in SMN protein levels. The SMN deficiency alters nuclear body formation and whether it can contribute to the disease remains unclear. Here we screen a series of small-molecules on SMA patient fibroblasts and identify flunarizine that accumulates SMN into Cajal bodies, the nuclear bodies important for the spliceosomal small nuclear RNA (snRNA)-ribonucleoprotein biogenesis. Using histochemistry, real-time RT-PCR and behavioural analyses in a mouse model of SMA, we show that along with the accumulation of SMN into Cajal bodies of spinal cord motor neurons, flunarizine treatment modulates the relative abundance of specific spliceosomal snRNAs in a tissue-dependent manner and can improve the synaptic connections and survival of spinal cord motor neurons. The treatment also protects skeletal muscles from cell death and atrophy, raises the neuromuscular junction maturation and prolongs life span by as much as 40 percent (p < 0.001). Our findings provide a functional link between flunarizine and SMA pathology, highlighting the potential benefits of flunarizine in a novel therapeutic perspective against neurodegenerative diseases.

## Introduction

Protein localization is critical for cellular functions and tissues homeostasis. Survival motor neuron (SMN) protein has a specific localization in the nucleus of eukaryotic cells^1–5^. It is found concentrated into the nuclear bodies Cajal bodies (CBs), which are hubs of small non-coding RNAs including the splicing small nuclear (sn)RNAs^6^. However, the role of SMN in CBs rem ains elusive. Altered CB localization of SMN protein is a hallmark of childhood spinal muscular atrophy (SMA) disease^2^, and of some other adult motor neuron disorders^7–9^. SMA is a hereditary neurodegenerative disease characterized by the death of spinal cord motor neurons and skeletal muscle atrophy. Mutations of SMN1 gene and the presence of SMN2 gene copies account for the deficiency in SMN protein responsible for SMA^10,11^, a leading cau se of infantile mortality. This deficiency occurs due to a specific alternative splicing of SMN2 gene^12–14^. Although the genetic basis of SMA is determined^10^, its neuromuscular manifestation and the mechanism underlying the disease severity are not fully understood.

SMN is a chaperone for the assembly of RNAs with their cognate ribonucleoproteins (RNPs)^15^. The best-characterized function of this ubiquitously expressed protein is a role in the snRNP biogenesis. The snRNPs are major components of spliceosomes that catalyse the removal of introns from pre-mRNAs to yield mRNAs^16^. SMN forms with Gemin 2 to 8 and unrip a multi-protein complex involved in the assembly of the Sm core proteins on snRNAs (U1, U2, U4, U5, U11, U12 and U4atac)^17,18^. Correlations exist between SMA disease severity, SMN protein levels^2,19^ and snRNP assembly capacity^20,21^. Severe SMN deficiency results in tissue- and snRNP-specific perturbations of snRNA levels in SMA mouse models^21,22^. Indeed, *in vitro* studies demonstrated that SMN regulates the relative abundance of individual snRNPs^23^. Although the precise role of aberrant splicing of specific genes in SMA is unclear, substantial evidence suggests that these changes might contribute to the disease severity^24–29^ thereby modulation of spliceosome machineries could have potential therapeutic application.

In recent years, several high-throughput screening studies have been carried out to identify small molecule modulators of general or alternative splicing in proliferating cells^30,31^. For example, one approach has shown that indole derivatives are potent splicing inhibitors with a selective action on splicing modulator SR proteins^32^. Another group has reported that the central nervous system (CNS)-penetrant drug flunarizine causes in HeLa cells splicing changes of numerous genes including intron retention in SMN and coilin genes, but the mechanism is unknown^33^. Another molecule has been identified that inhibits *in vitro* the splicing of multiple pre-mRNAs and causes CB disruption and SMN aggregates in the cytoplasm of HeLa cells^34^. However, these molecules have not been investigated *in vivo* in splicing disease models. Other screening assays have identified small molecules that specifically modulate the splicing of transcripts from SMN2 gene to compensate for the lost SMN1 gene and improve the symptoms of SMA mouse models^35^. Because some of these drugs are likely to have side effects with long-term usage, the combination of two or more molecules, which target distinct facets of SMA cell biology, at low dosage might be a more relevant therapy^36^.

To further study SMN localization into nuclear bodies, we screened small-molecules using a microscopy cell-based assay to detect SMN into CBs of SMA patient-derived fibroblasts, identified flunarizine as a positive hit, and evaluated the effects of the drug in a mouse model of SMA. We found *in vivo* with flunarizine treatment the enrichment of SMN into CBs of spinal cord motor neurons and tissue-specific modulation of the splicing snRNA levels in SMA mice. Moreover, we demonstrated that treatment with flunarizine improves the synaptic alterations of spinal cord motor neurons and muscle phenotype, and expands survival of SMA mouse models. We conclude that flunarizine represents a potential therapeutic strategy, alone or in combination, to reduce the disease severity of SMA.

## Results

### Cell-based screening of SMN protein in CBs identifies small-molecules

**Figure 1:**
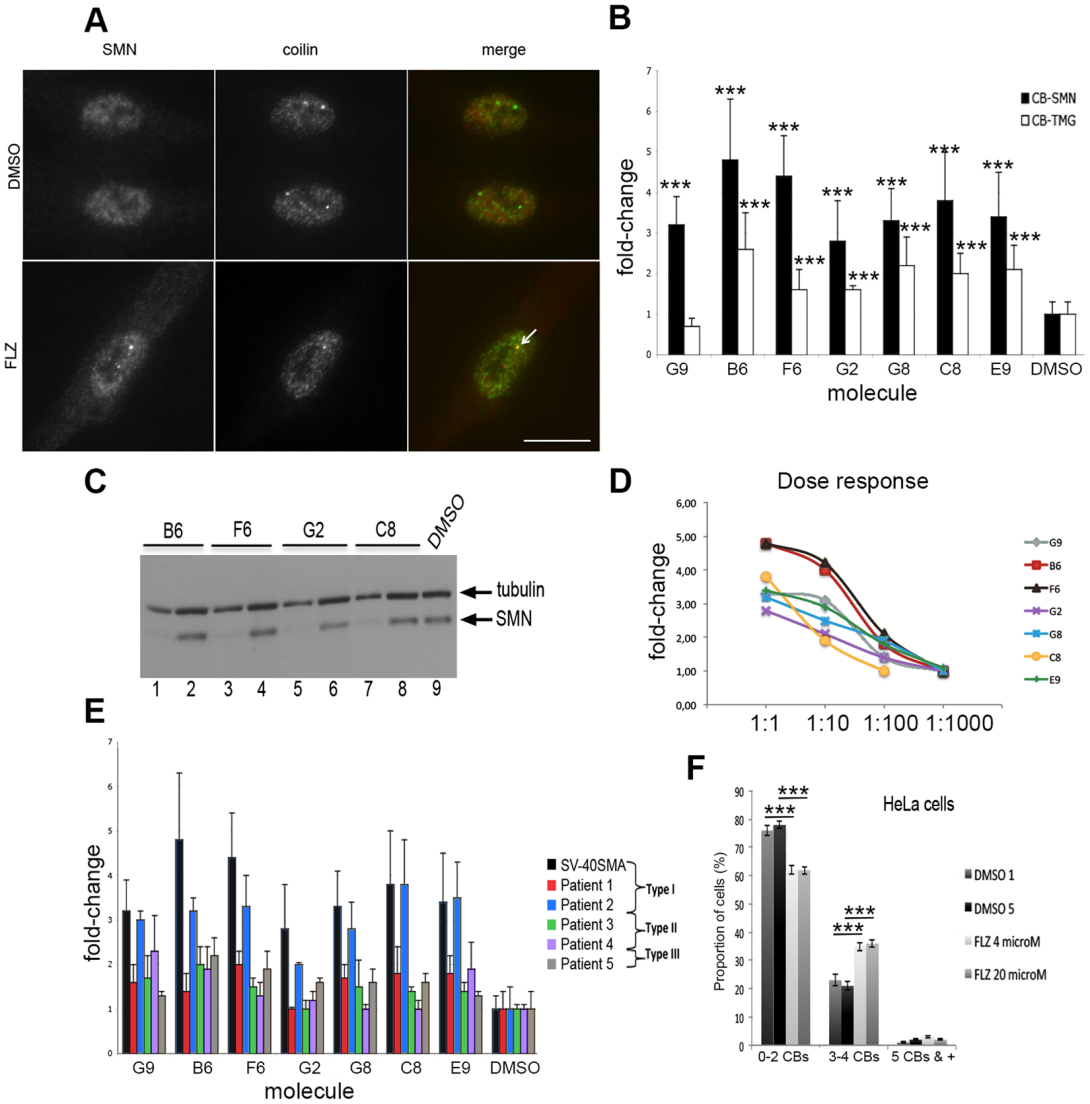
Microscopy cell-based assay to screen for SMN protein in nuclear-body Cajal bodies (CBs) of SMA patient-derived fibroblasts. (**A)** Immunofluorescent staining of SMN and CB-marker coilin protein in flunarizine treated (2 μg/ml) and untreated (DMSO) immortalized Type I SMA fibroblasts using rabbit polyclonal anti-SMN and mouse monoclonal anti-coilin antibodies. (**B)** Analysis of the immunolocalization of SMN and snRNPs (TMG-capped snRNAs) into CBs of immortalized Type I SMA fibroblasts for seven molecules. (**C)** Total protein extracts from immortalized type I SMA fibroblasts treated with 2 μg/ml of each molecule were separated by SDS-PAGE and SMN protein was detected by western blotting using tubulin as loading control. Odd and even numbers correspond to 5 and 15 μg of proteins, respectively. (**D)** The ability of the molecule to recruit SMN to CBs in immortalized SMA fibroblasts was tested in the presence of decreasing concentrations of each molecule, the 1:1 dilution being 2 μg/ml. (**E)** Analysis of the immunostaining of SMN in CBs of drug-treated primary fibroblast cultures from patients affected with the three forms of SMA disease. See Supplemental Table 3 for statistical analyses. (**F)** HeLa cancer cells were treated with 4 (2 μg/ml) and 20 μM flunarizine for 4 h and compared to 1 or 5 μl/ml DMSO. An increase in the proportion of cells with 3 or 4 CBs per nucleus is observed with both concentrations of flunarizine. Data represent the mean values ± SEM. Chi2 test, ***p < 0.001. Scale bar, 10 μm.

We developed and carried out an immunofluorescence microscopy assay using an SV40 large T antigen immortalized Type I SMA patient fibroblast cell line previously described^37^. The screening was based on double immunodetection of SMN and CB-marker coilin proteins (Fig. 1A). Due to the high nucleoplasm immunolabeling, we manually analysed the effects of 244 compounds on the CB localization of SMN. The primary screening of a 16 h exposure, at the concentration of 2 μg/ml, showed ≈40 molecules with an effect of more than 2.5 times the number of cells with SMN-positive CBs compared to DMSO treated cells (Chi2, p < 0.0001, 300 cells, 3 independent experiments). Some compounds might have a transient effect, we therefore counter-screened the same compounds for a 72 h exposure with one dose at the beginning of the experiments. Nine molecules passed the selectivity step of which one was no longer available (Pindobind-5HT1A) and another one was the hydrolyzed product of one of the nine compounds (uridine 5′-diphosphate versus triphosphate salt). Seven molecules were therefore positive hits in our assay (Fig. 1B, Supplemental Table 1). We also determined the effect on the CB localization of the splicing snRNA using antibodies against the trimethylguanosine (TMG)-capped snRNAs (molecular signature for the nuclear import of snRNAs). The highest fold-change in TMG-labelling of CBs was observed with B6 (flunarizine) in Fig. 1B. We then investigated the SMN protein levels in protein extracts from treated immortalized SMA fibroblasts by western blotting (Fig. 1C). No significant effects on the SMN protein levels were revealed with flunarizine at 2 μg/ml. Moreover, measurements of four dilutions (2 μg/ml, 200 ng/ml, 20 ng/ml and 2 ng/ml) in our immortalized SMA cell model showed a dose-dependent accumulation of SMN to CBs with flunarizine (B6) and gabapentine (F6; Fig. 1D). The half maximal concentration (EC50) was determined for each compound (Supplemental Table 2). Flunarizine (B6) and gabapentine (F6) showed the best fold induction at EC50 with concentrations of 80 and 200 nM, respectively. We then examined the effect of the molecules in primary cultures of patient-derived fibroblasts from all three forms of SMA disease (Fig. 1E). A two-fold induction in SMN to CBs was also shown with flunarizine on primary cultures derived from Type 2 and Type 3 SMA patients. Statistical analyses revealed that significant effects are observed for flunarizine in all SMA cells, excepted with the Coriell type I SMA patient fibroblast line #GM03813 (Supplemental Table 3). In another study, it has been shown that treatment of HeLa cells with flunarizine (FLZ, 20 μM) for 4 h changes the splicing expression pattern^33^. We performed immunofluorescence experiments with HeLa cells using flunarizine at doses of 4 (2 μg/ml) and 20 μM (10 μg/ml) for 4 h (Fig. 1F). Both concentrations increased the proportion of cells with more SMN-positive CBs compared to DMSO-treated cells (Chi2, p < 0.001). We therefore tested *in vivo* whether flunarizine could have protective effects in an SMA mouse model.

### Flunarizine improves survival, body weight and motor deficits of SMA mice

**Figure 2:**
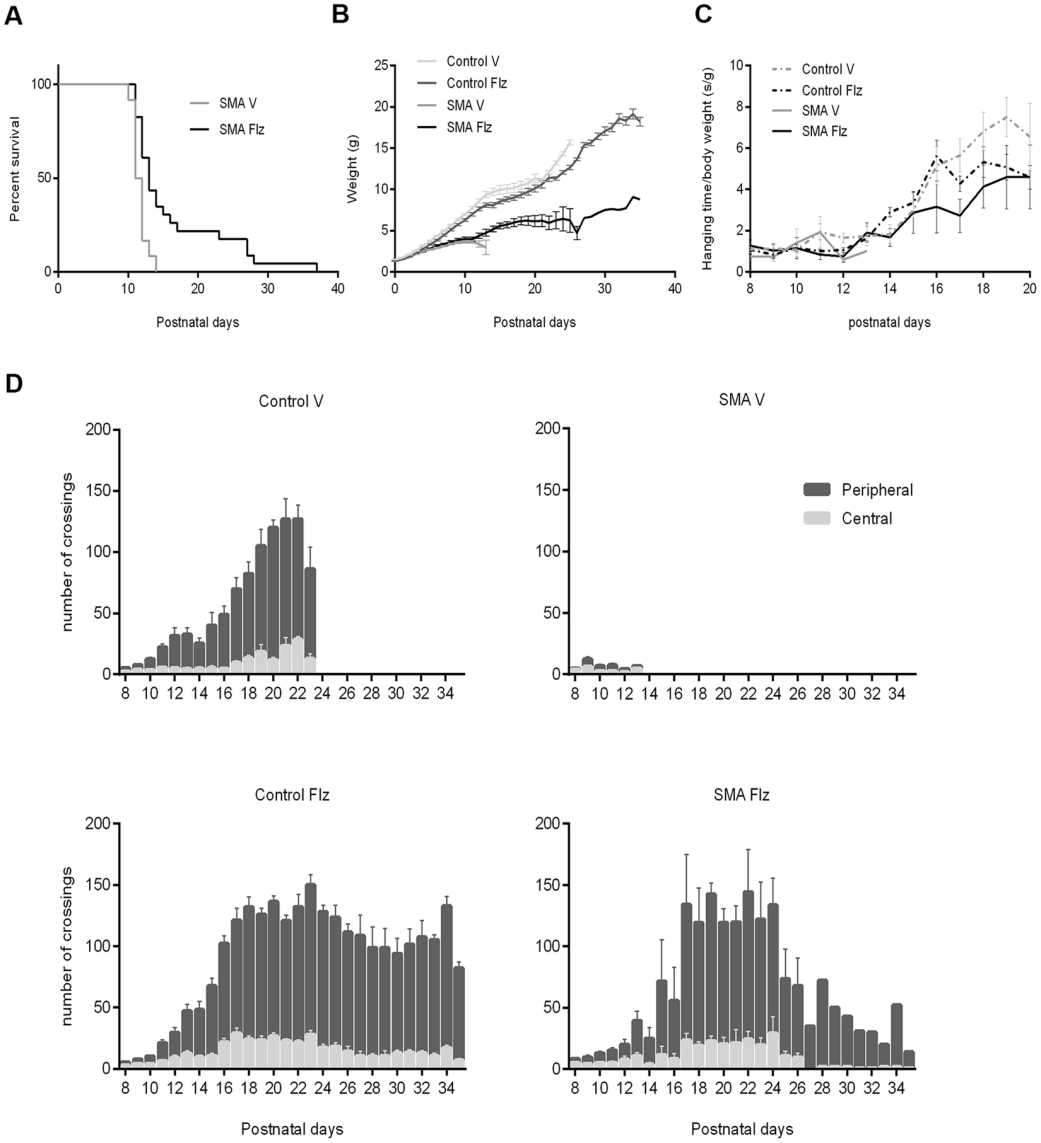
Treatment with flunarizine improves the phenotype of SMA mice. (**A**) Kaplan-Meier survival curves for vehicle (V)- (n = 19) and flunarizine (Flz)-treated SMA mice (n = 12); (**B**) Body weight of vehicle- (n = 67) and flunarizine-treated controls (n = 68) and vehicle- (n = 19) and flunarizine-treated SMA mice (n = 12); (**C**) antigravity hanging performance of vehicle- (n = 35) and flunarizine-treated controls (n = 40) and vehicle- (n = 18) and flunarizine-treated SMA mice (n = 10); (**D**) Number of crossings during 5 min for vehicle- (n = 35) and flunarizine-treated controls (n = 40) and vehicle- (n = 19) and flunarizine-treated SMA mice (n = 10). The drug is administrated at a daily dose of 0.5 mg/kg. Mean ± SEM.

The *in vivo* studies were performed with the severe Taiwanese SMA mouse model (Smn^ko/ko^; SMN2^tg/0^) and control heterozygous (Smn^ko/wt^; SMN2^tg/0^) mice^38^. From birth until the death of SMA mice, flunarizine was administrated daily by intrathecal injections for rapid effects at doses of 0.125 (5 SMA, 10 controls), 0.25 (5 SMA, 8 controls) and 0.5 mg/kg (12 SMA, 31 controls) in a pilot study to evaluate survival. Due to the poor solubility of flunarizine, 0.5 mg/kg was the highest concentration used. The two lowest doses did not have significant effects on survival of SMA mice (data not shown). Additional 16 SMA mice (11 with 0.5 mg/kg flunarizine and 5 with vehicle) were studied in a blinded manner and included in the analysis (Fig. 2A). The SMA mice treated with vehicle died at about post-natal day 12 (P12). The flunarizine treatment significantly extended lifespan of ≈40% with a shift in mean survival of SMA mice (16.3 versus 11.7 days; Mantel-Cox test, p = 0.0002). One SMA mouse lived 36 days and was euthanatized. These findings indicate that flunarizine impacts survival of SMA mice.

The body weight (BW) was the first sign of phenotypic difference between vehicle-treated SMA and control mice that became apparent at P5 (Fig. 2B) and the difference increased with age because of the continued gain of BW in controls (2-way ANOVA, p < 0.001). There is a significant difference of BW between the vehicle- and flunarizine-treated controls between P4 and P15, and after P23. The flunarizine-treated SMA mice showed a modest sustained gain of BW, reaching a plateau at ≈P18. Antigravity hanging and open field tests were performed starting at P8 to evaluate the muscle strength of the forelimbs and the basal locomotion function of SMA mice, respectively^39^. The flunarizine-treated SMA mice performed similarly to controls in the antigravity hanging assay (Fig. 2C, 2-way ANOVA + Tukey’s, not significant). All four groups of mice showed little mobility in the open field between P8 and P10 (Fig. 2D). From P12 (when autonomous walking has taken place), the vehicle and flunarizine-treated control mice showed an increased activity, travelling and crossing a larger number of squares. The vehicle-treated SMA mice showed little ambulatory movements. Flunarizine clearly improved mobility of mutants as shown by an increased number of crossings similar to controls until P23, excepted at P16 for the distance travelled within the center. These data indicate that flunarizine corrects the hypoactivity of SMA mice.

### SMN in Cajal bodies of spinal cord motor neurons increases with flunarizine in SMA mice

**Figure 3:**
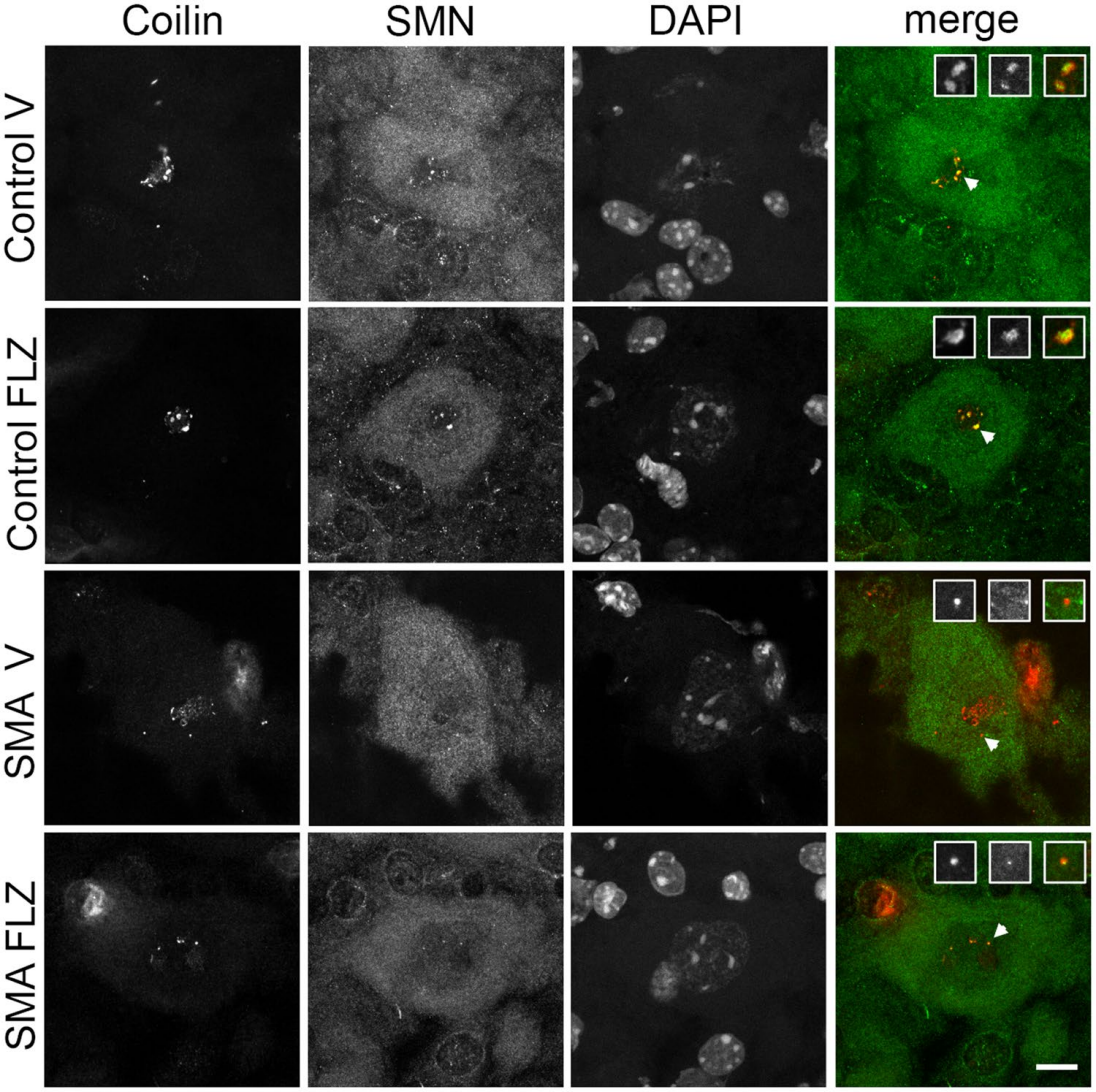
Treatment with flunarizine accumulates SMN into nuclear-body Cajal bodies in spinal cord motor neurons in SMA mice. Immunofluorescent staining was to detect SMN and Cajal bodies in lumbar spinal cord motor neurons of 11 day-old mice using rabbit monoclonal anti-SMN and mouse monoclonal anti-coilin antibodies. The confocal microscope was focused on coilin-positive Cajal bodies. The laser was adjusted to obtain similar labeling intensities. Scale bar, 10 μm.

We thus examined the effects of flunarizine on the localization of the SMN protein in nuclear bodies of spinal cord motor neurons of mice at P11. By immunofluorescent labeling of lumbar spinal cord cross-sections with anti-coilin and anti-SMN antibodies, we observed that motor neurons of the vehicle-treated SMA mice displayed as many CBs as controls (Fig. 3A, Table 1). Whereas the vehicle-treated mutants exhibited fewer SMN-positive CBs (0.4 ± 0.2) than controls (1.8 ± 0.1), the flunarizine-treated mutants showed a significant 2-fold increase of SMN-positive CBs in motor neurons (Chi2 1ddl, p«0.001). SMN is also present in other nuclear bodies, the gems that are devoid of coilin^1^. Gems are reduced in motor neurons of vehicle-treated mutants (0.3 ± 0.2) compared to controls (0.5 ± 0.2), whereas they increased in flunarizine-treated mutants (0.6 ± 0.1; Chi2 1ddl, 0.02 < p < 0.05). Our findings indicate that flunarizine localizes SMN into nuclear bodies of motor neurons in SMA mice.

**Table 1:** Localisation of the SMN protein in nuclear-body Cajal bodies (CBs) and Gems in spinal motor neurones (MNs) of FLZ-treated SMA mice.

	Number of animals (MN number)	Coilin-positive nuclear bodies (CBs) per MN	SMN-positive CBs per MN	Gems that are not in CBs per MN
Control-vehicle	3 (177)	2.9 ± 0.03	1.8 ± 0.1	0.5 ± 0.2
Control-FLZ	3 (351)	3.3 ± 0.1	2.0 ± 0.2	0.5 ± 0.1
SMA-vehicle	3 (118)	3.3 ± 0.4	0.4 ± 0.2	0.3 ± 0.1
SMA-FLZ	5 (215)	2.6 ± 0,2	0.8 ± 0.2	0.6 ± 0.1

**Figure 4:**
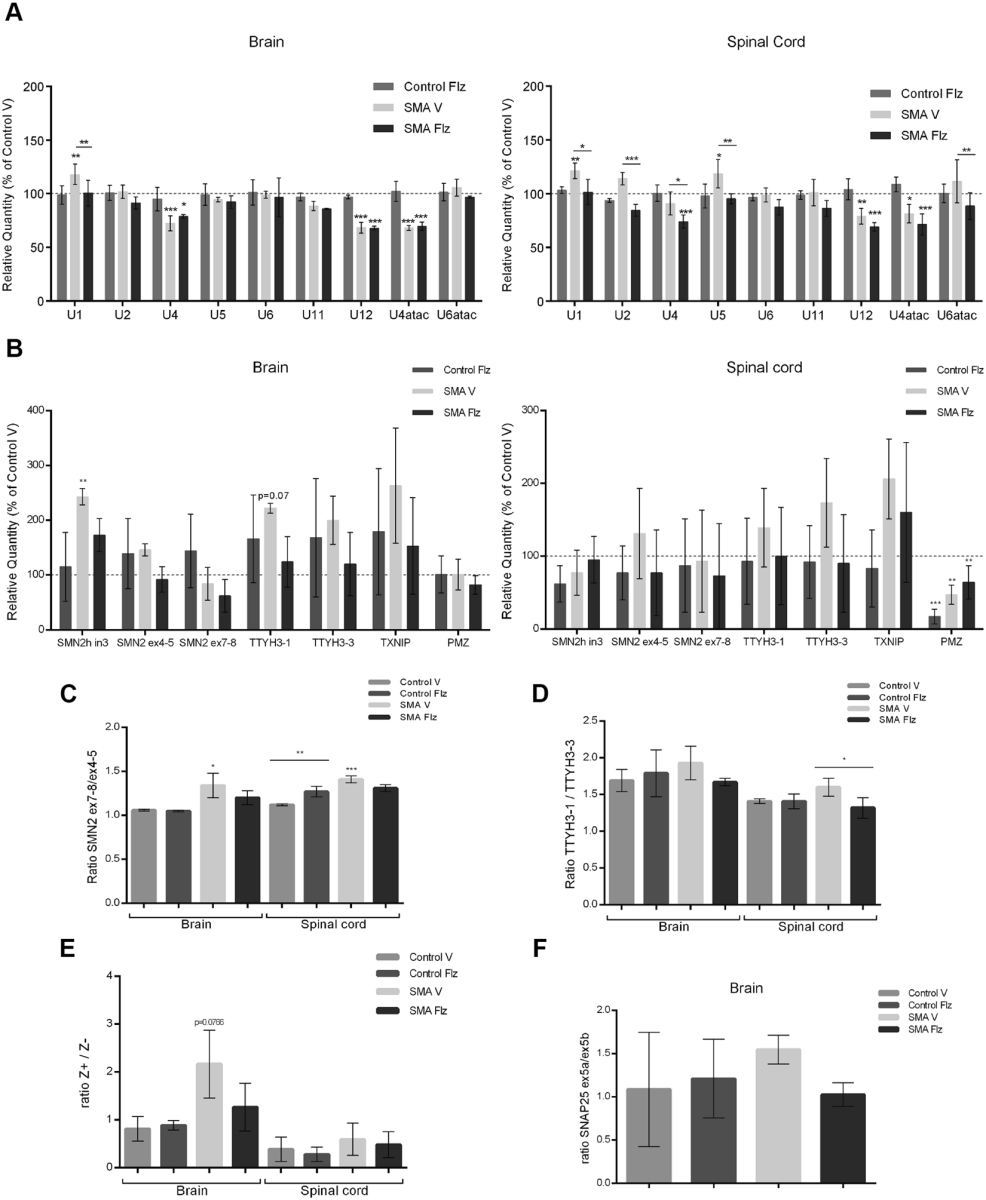
Effects of flunarizine on the expression and splicing profiles of candidate genes in SMA mice. (**A**) The snRNP-specific reduction of snRNAs in brain and spinal cord of SMA mice is modulated by flunarizine. The snRNA levels are determined by RT-qPCR and the relative amount is presented as percent of the vehicle-treated controls. 5 S and 5.8 S were used as controls for normalization as described^22^. (**B**) RT-qPCR analysis of different genes shows that flunarizine reduces the retention of SMN2 intron3 in brain of SMA mice. Four genes were used as controls for normalization in brain (RPL13A, PPIA, HPRT1, SDHA) and spinal cord (RPL13A, PPIA, HPRT1, ACTB). (**C**–**F**) The expression ratio between exons or isoforms of SMN2, TTYH3, AGRN and SNAP25 genes. SMN2 and TTYH3 were analysed by RT-qPCR whereas AGRN and SNAP25 were by RT-PCR experiments. No significant differences of SMN2 exon 7/ex 4 ratios were found between flunarizine- and vehicle -treated SMA mice in panel 4 C (p = 0.007). Total RNA was prepared from tissues of flunarizine (Flz)- and vehicle (V)-treated controls and SMA mice at P11. Three mice per group. Data represent the mean values ± SD (errors bars). Statistical test used is the two-way Anova followed by the Tukey’s multiple comparisons test (GraphPad).

We next evaluated by RT-qPCR the spliceosomal snRNA levels in total RNA preparations from brain and spinal cord of flunarizine- and vehicle-treated mice at P10 (Fig. 4A). Using previously described primers^22,25^, similar reduction in snRNA levels were observed in SMA mice, including the minor spliceosomal U12, which was decreased by about 20 to 30% in the brain and spinal cord, as compared to the corresponding controls. The treatment significantly modulated the levels of U2, U4, U5 and U6atac in the spinal cord and of U1 snRNA in the brain and spinal cord of SMA mice. Our results suggest that flunarizine might regulate the relative ratio between snRNAs.

We then explored mRNA changes in brain and spinal cord of SMA mice (Fig. 4B–F). We analysed SMN2 exon7 inclusion and intron3 retention, the later being influenced by flunarizine in HeLa cells^33^. We observed no significant effects of flunarizine on the SMN2 expression levels (exon 4–5) or exon7 inclusion in SMA mice (Fig. 4B,C). Of note, SMN2 intron3 levels increased in brains of SMA mice whereas they decreased with flunarizine. We analysed other genes being influenced by flunarizine in HeLa cells namely TTYH3, TXNIP, PMZ and SNAP25 (Fig. 4B,D,F). No significant differences were detected with flunarizine in SMA mice, excepted for the relative expression levels of the 5′end versus the 3′end of TTYH3 in the spinal cord of SMA mice (Fig. 4D). Moreover, PMZ levels decreased in spinal cord of SMA mice compared to controls (Fig. 4B). The neuronal Z^+^AGRN isoforms were expressed in both tissues of SMA mice (Fig. 4E). Our results differ from that reported with HeLa cells, suggesting that the effect of flunarizine depends on cell type.

### vGlut1 synapses on spinal cord motor neurons of SMA mice increase with flunarizine

**Figure 5:**
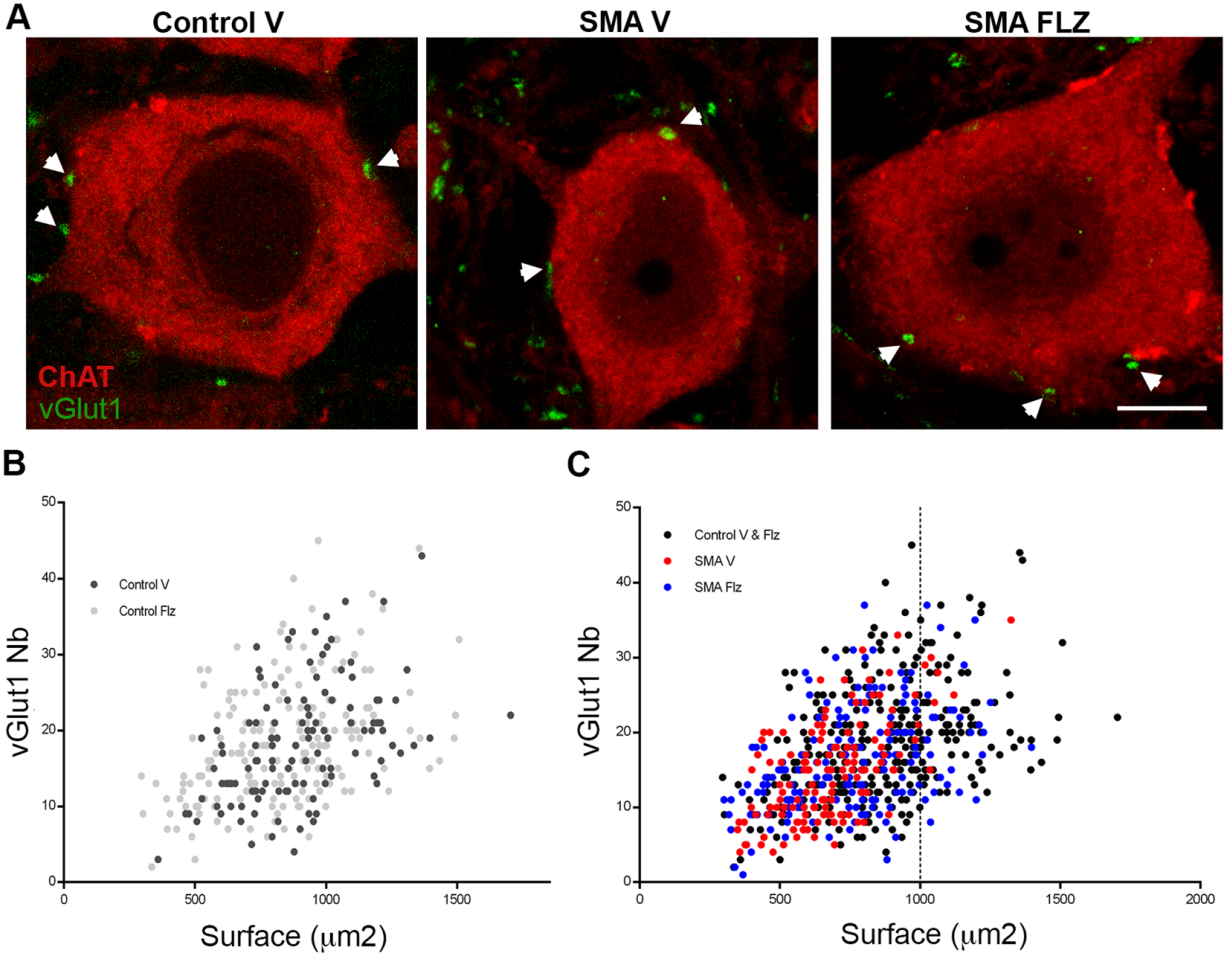
Treatment with flunarizine elicits protective effects on spinal cord motor neurons of SMA mice. (**A)** Co-labeling of the glutamatergic synapse marker vGlut1 (green, arrowheads) and the motor neuron marker ChaT (red) to assess the central excitatory glutamatergic synapses on spinal cord motor neurons of controls and SMA mice in response to flunarizine. Scale bar, 10 μm**. (B)** Analysis of vGlut1 appositions per motor neuron of flunarizine (Flz)- and vehicle (V)-treated control mice. Each motor neuron is represented with a dot and the number of vGlut1 boutons is plotted as a function of the somata size. There is no difference between the two control groups. (**C)** Distribution of the spinal cord motor neurons of flunarizine- (bleue dots) and vehicle-treated SMA mice (red dots) compared to all controls from panel B (black dots). The treatment increases both the frequency of motor neurons with 24 to 30 vGlut1 boutons and the number of larger motor neurons (≥1000 μm) in SMA mice.

Spinal motor neurons of SMA mice show a reduced number of synapses positive for the vesicular glutamate transporter 1 (vGlut1), a selective synaptic marker of proprioceptive primary afferents^40^. We examined those synapses in lumbar spinal cord cross-sections of vehicle and flunarizine-treated mice at P10 by co-immunolabeling of motor neurons with anti-choline acetyltransferase (ChAT) and anti-vGlut1 antibodies (Fig. 5A). We analysed the fluorescence images of 118 control and 136 SMA motor neurons from vehicle-treated mice and 183 control and 171 SMA motor neurons from flunarizine-treated mice. We excluded the motor neurons with a soma size less that 300 μm^2^ because there are mostly gamma motor neurons with few or no vGlut1-positive afferents^41^. We determined the number of vGlut1 synapses per motor neuron (y axis) and the cross-sectional size of motor neurons (x axis, Fig. 5B). No significant differences were found in motor neurons of vehicle and flunarizine-treated control mice (Kruskal-Wallis + Dunn’s multiple comparisons test, NS p > 0.9999). The number of vGlut1 synapses and the size of spinal cord motor neurons were both reduced in vehicle-treated SMA mice (red dots, p = 0.0023) compared to controls (black dots, Fig. 5C). We found motor neurons (blue dots) with large soma size and increased vGlut1 boutons in all cell populations of flunarizine-treated SMA mutants at level similar to controls (p = 0.1254). Moreover, a 3-fold increase in the number of large motor neurons (≥1000 μm) was observed in flunarizine-treated SMA mice. Our results indicate that flunarizine protects spinal cord motor neurons of SMA mice.

### Flunarizine modulates the expression of fiber type IIa in SMA mouse skeletal muscles

Biondi *et al*. previously showed an altered muscle typology in SMA mice^42^. We asked whether flunarizine could modulate those changes. In skeletal muscles, fibers are designated by the myosin heavy chain (MyHC) isoform expressed, namely type I and type II for slow and fast myosin respectively. Moreover, the type II fibers sub-divide into IIa, IIb and IIx/d according to their oxidative and glycolytic capacities. Individual muscle has a defined proportion of fiber types^43,44^. Two additional isoforms are expressed during post-natal development. The neonatal MyHC (Neonat) is a marker of postnatal maturation whereas the embryonic MyHC (Emb) persists for a time after birth. We used a panel of specific-MyHC antibodies to explore three hind-limb muscles, the soleus, plantaris and tibialis in vehicle- and flunarizine-treated control and SMA mice at P11. The soleus is the less mature muscle, as indicated with a larger proportion of embryonic fibers than in the plantaris and tibialis. Their proportions significantly decreased in the soleus and tibialis of flunarizine-treated SMA mice compared to vehicle-treated mutants (Fig. S1A). The proportion of type I fibers in the soleus of vehicle-treated SMA mice was larger than in controls and flunarizine-treated mutants. Moreover, type IIa fibers significantly increased in the soleus and plantaris of flunarizine-treated mutants whereas the soleus of vehicle-treated mutants was missing about one-third of type IIa fibers compared to controls.

**Figure 6:**
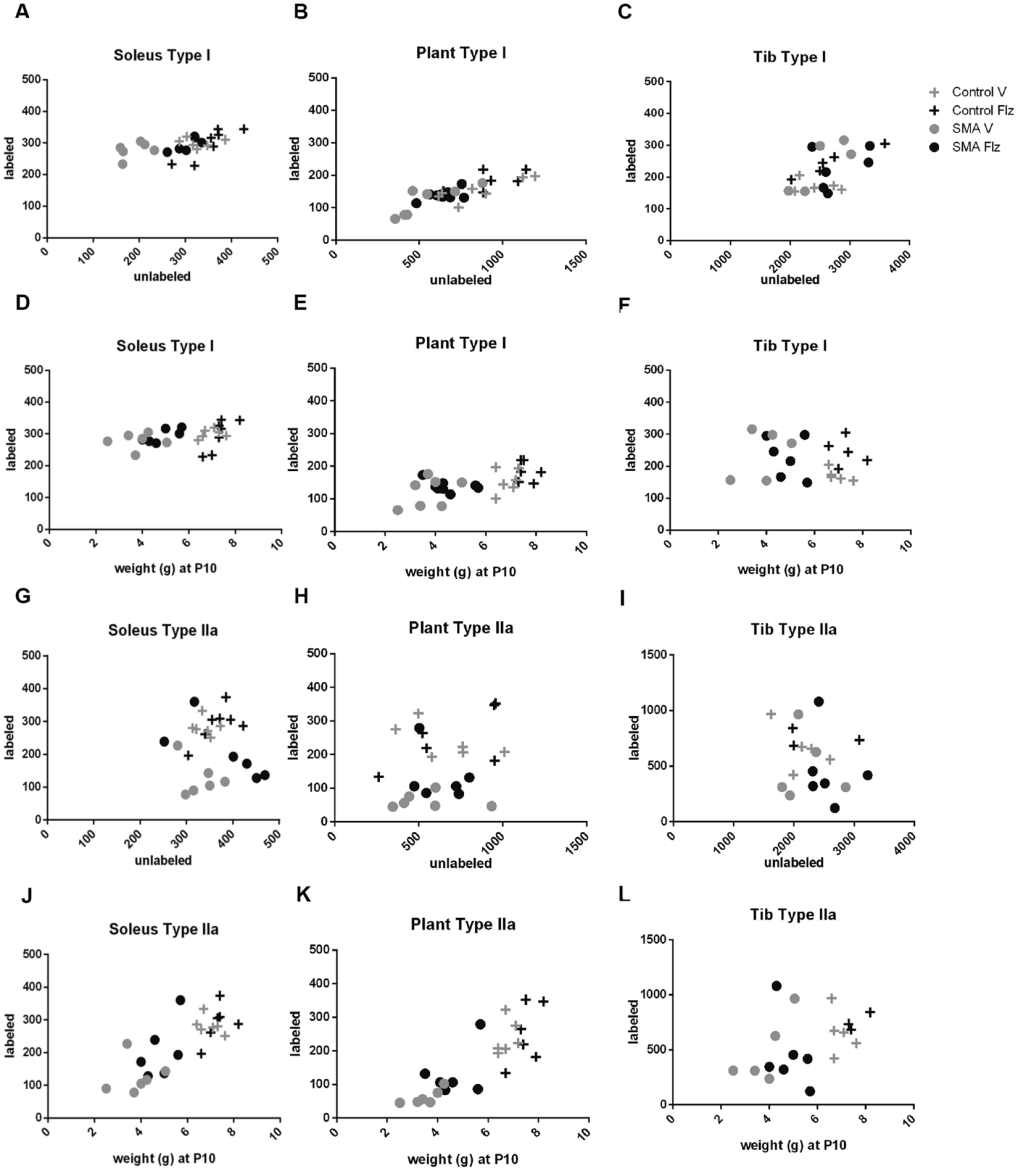
Differential changes in slow and fast muscle fibers of SMA mice treated with flunarizine. Type I and type IIa muscle fibers compose the slow and fast fatigable motor units, respectively. Myofiber composition is determined by the presence of specific myosin heavy chain (MyHC) isoforms: type I being coded by the MYH7 gene and type IIa by MYH2 gene. The fiber types are revealed by immunofluorence experiments using specific anti-MyHC antibodies. The graphs show the number of immunolabeled type I or type IIa fibers (y axis) versus unlabeled fibers or body weight (x axis) for the soleus, plantaris and tibialis of 10-day-old mice for n ≥ 5 mice per experimental group excepted for the tibialis from control mice (n = 3). Each mouse is presented with a symbol. A fiber type shift takes place in SMA mice treated with flunarizine (Flz), as detected by a shift of type IIa positive fibers in the soleus and plantaris of SMA mice. See also the Supplemental Fig. 1 for frequency histograms of type I, II, IIa, neonatal and embryonic myofibers.

We set up additional analyses to further characterize the changes among the four groups of mice (Fig. 6): 1) a comparison between the number of immuno-labeled (y axis) and unlabeled (x axis) fibers (Fig. 6A–C, G–I) and 2) a comparison between immuno-labeled fibers (y axis) and the BW (x axis) in Fig. 6D–F, J–L. Using the comparison with either labeled and unlabeled type I or type IIa fibers, in both the soleus and plantaris we observed differences based on the mouse status (controls versus SMA) and on the treatment (vehicle versus flunarizine) for unlabeled type I (Fig. 6A,B) and labeled type IIa fibers (Fig. 6G,H). The differences among the four groups were highly significant in the soleus for unlabeled type I (Kruskal-Wallis Chi2 (3ddl) p = 0.0014) and type IIa fibers (p = 0.0025), and in the plantaris, for type IIa fibers (p = 0.0011). In the soleus of SMA mice, the effect of flunarizine was significant with unlabeled type I fibers, which are presumably type II fibers, (1ddl, p = 0.0039) and almost significant for type IIa fibers (p = 0.0547). In the plantaris of SMA mice, a similar effect of the drug was detected with type IIa fibers (1ddl, p = 0.0104). We also asked whether a correlation exists between MyHC-labeled fibers and BW (Fig. 6D–F, J–L). A significant effect was detected for type IIa fibers and BW in the soleus (r = 0.7843 p < 0.01, Fig. 6J) and in the plantaris (r = 0.8388, p < 0.01) whereas no correlation was shown for type I fibers and BW. The results with the embryonic fibers in the soleus were similar to those with type I fibers (Supplemental Fig. 1B). For the tibialis, no significant differences were detected with both unlabeled type I and labeled type IIa fibers (Fig. 6C,F,I and L). Our results support the view that type IIa fiber deficiency of SMA mice is a disease feature partially rescued with flunarizine.

### Fiber size and number are increased by flunarizine in a muscle-dependent manner in SMA mice

**Figure 7:**
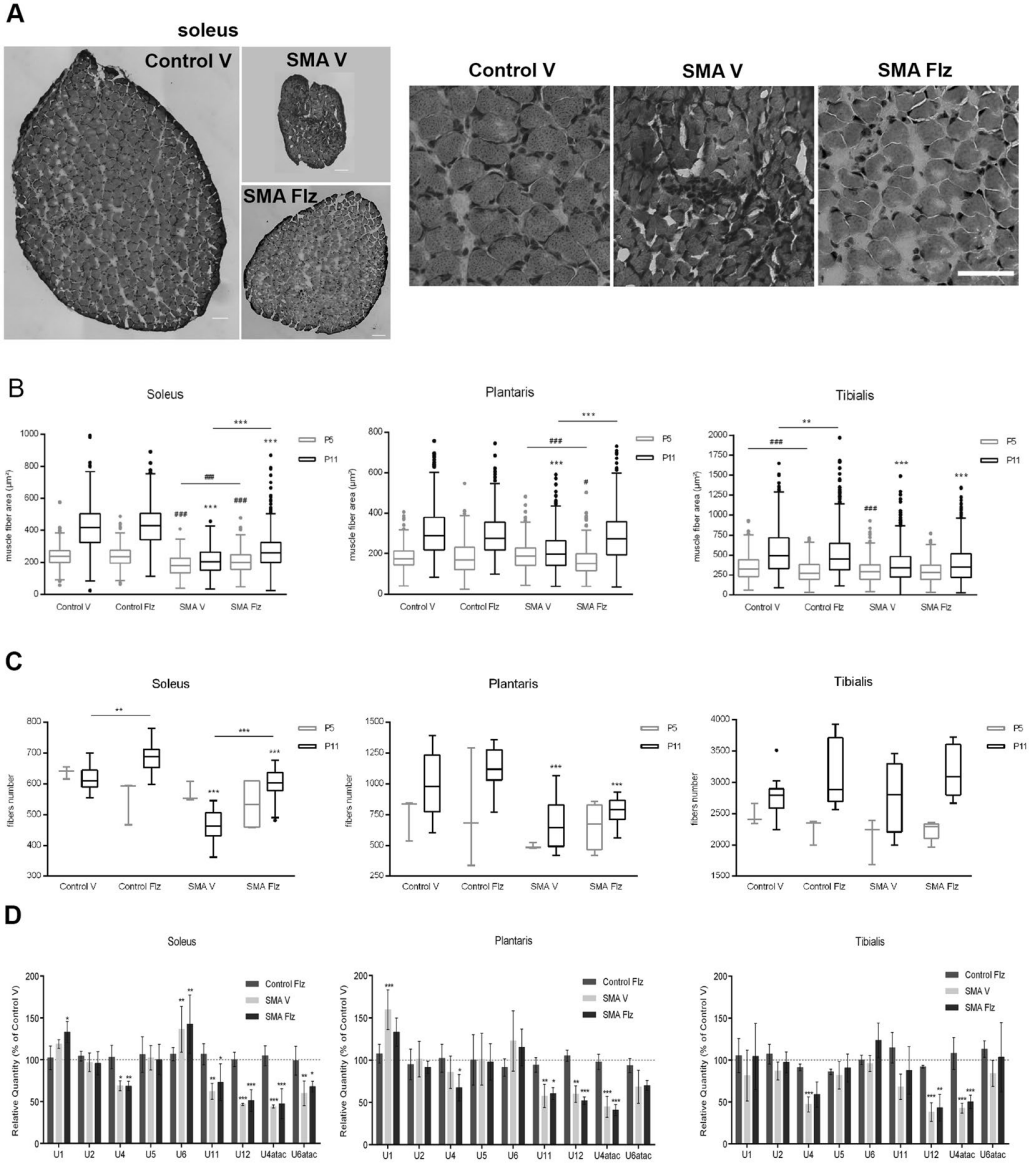
Muscle morphology reveals muscle-specific responses to treatment with flunarizine in SMA mice. (**A)** Hematoxylin and eosin staining of the soleus from 11-day-old vehice-treated control mouse compared to vehicle- and flunarizine-treated SMA mice. Scale bar, 50 μm. (**B)** Analyses of the fiber size in the soleus, plantaris and tibialis of flunarizine- and vehicle-treated control and SMA mice. Data represent fiber area ± SEM (errors bars) of three (5-day-old, in grey) to ≥5 mice (11-day-old, in black) per experimental group. The flunarizine treatment corrects the atrophy in the plantaris and reduces it in the soleus of SMA mice. The statistical analysis was performed using Kruskal-Wallis test followed by Dunn’s multiple comparison rank test. Wiskers are calculated by the Tukey’s method. The * and # between 5- and 11-day-old mice, respectively. One to three symbols represent p < 0.1, 0.01 and 0.001, respectively. (**C)** Analyses of the number of fibers for the three muscles for the same number of mice per group. The treatment prevents the loss of fibers in the soleus of SMA mice. (**D)** The snRNP-specific reduction of snRNAs is observed in the soleus, plantaris and tibialis of SMA mice. Total RNA was prepared from tissues of flunarizine- and vehicle-treated controls and SMA mice at 11 days of age (3 mice per group). The snRNA levels are determined by RT-qPCR and the relative amount is presented as percent of the vehicle-treated controls. Statistical analyses are performed as in Fig. 3. The treatment with flunarizine has modest effects on snRNA levels in muscles of SMA mice.

Both the size and number of muscle fibers are reduced in SMA mouse models. Cross-sections of the soleus, plantaris and tibialis were stained with hematoxylin and eosin (H&E) to determine the size of the fibers in vehicle- and flunarizine-treated mice at P5 and P11 (Fig. 7). A therapeutic window between P5 and P8 has been documented in SMA mouse models^45,46^. We confirmed that fibers are smaller in individual SMA muscle than in controls at P11, and in the soleus and tibialis at P5 (Fig. 7B). The atrophy is more severe in the soleus than in the plantaris and tibialis. Upon treatment with flunarizine, significant differences were no longer observed between the plantaris of mutants and controls. A detailed analysis revealed that flunarizine reduced the proportion of smaller fibers in the soleus and restored the relative fiber size distribution of the plantaris (Fig. S2). We next determined the number of fibers in individual muscles in mice at P5 and P11 (Fig. 7C). We observed at P5 no reduction of fiber number in individual muscle of vehicle-treated mutants compared to controls, whereas at P11 a reduction was confirmed for the soleus (25% less) and plantaris (40% less). No differences on the fiber number in the tibialis were seen among animals. At P11, the flunarizine treatment reversed the changes in the soleus of mutants, whereas no significant effects on the fiber number were observed in the plantaris. Our findings reveal the beneficial effects of flunarizine on individual SMA muscle during post-natal development.

We also evaluated the snRNA levels in the muscles of SMA mice (Fig. 7D). As reported earlier^22^, we observed that the minor spliceosomal snRNAs were markedly reduced in SMA mice, U12 and U4atac being decreased by about 40 to 60% in the three muscles. Flunarizine had no or modest effects on the snRNA levels in muscles, suggesting that the muscle improvements correlate with the neuroprotective effects of flunarizine.

### The maturation of neuromuscular synapses is promoted by flunarizine in SMA mice

**Figure 8:**
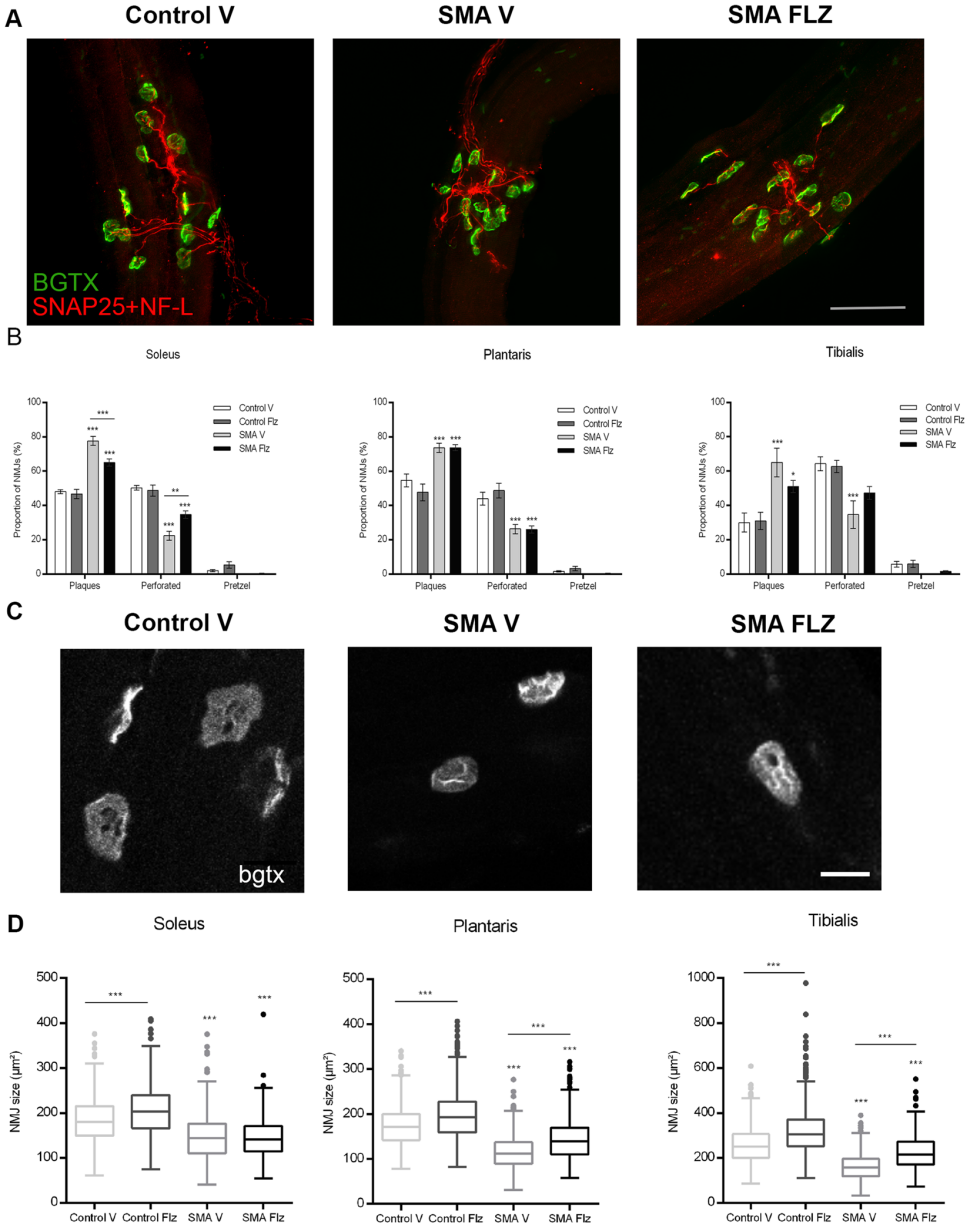
Flunarizine mitigates the defects at the neuromuscular junctions of SMA mice. (**A)** Immunofluorescent analyses of the neuromuscular junctions (NMJs) in the tibialis using anti-SNAP25 and anti-neurofilament light chain antibodies in red and labeled alpha-bungarotoxin (BGTX) staining for the acetylcholine receptors (AChRs) in green for the pre- and postsynaptic NMJs, respectively. Scale bar, 100 μm. (**B)** Frequency histograms depict the proportion of neuromuscular junctions in three stages (plaques, perforated structures and pretzels) in three muscles, namely soleus, plantaris and tibialis. The analysis shows that endplates are more elaborated in the soleus and tibialis of SMA mice upon the flunarizine treatment. Statistical test is two-way ANOVA followed by Tukey’s multiple comparisons test. Three mice per group. (**C)** Representative motor endplates from the tibialis of controls and SMA mice. Scale bar, 15 μm. (**D)** Quantification of the areas of the AChR clusters reveals a significant increase in endplate size in control and SMA mice upon flunarizine treatment. Kruskal-Wallis followed by Dunn’s multiple comparison rank test. Wiskers are calculated by the Tukey ‘s method. Three mice per group.

Structural alterations of the neuromuscular junctions (NMJs) are other hallmarks of SMA disease^47,48^. The acetylcholine receptors (AChRs) at the muscle plasma membrane form in embryos aggregates (plaques) that become perforated and mature to finally give to the NMJs a pretzel-like shape during post-natal development. We examined the NMJs in the soleus, plantaris and tibialis of vehicle- and flunarizine-treated SMA and control mice at P10 (Fig. 8). Immunofluorescence experiments were performed with longitudinal fibers using labeled alpha-bungarotoxin (BGTX) that binds specifically to the AChRs and antibodies against neurofilaments and the synaptic vesicle protein SNAP25 to decorate the motor neuron terminals. We confirmed that this SMA mouse model shows no denervation of the hindlimb muscles (soleus, plantaris and tibialis)^49^. We also confirmed that mutants have significantly more NMJs without a perforation (plaques) in individual muscles compared to controls, the tibialis being the most mature muscle. Indeed, in mutant muscles >65% of NMJs remain as plaques whereas <50% are found in controls. Flunarizine significantly increased the maturation of the NMJs in the soleus and tibialis of SMA mice, as indicated by approximately 15% increase of perforated structures (Fig. 8B). We then examined the size of the NMJs. They were smaller in mutants than in controls and increased in size in the plantaris and tibialis with flunarizine (Fig. 8C,D). The relative distribution of NMJ area showed that flunarizine shifted the distribution towards larger NMJs in all conditions. Our data reveal that flunarizine promotes the NMJ maturation in SMA mice.

## Discussion

SMN protein deficiency is responsible for the infantile neurodegenerative motor neuron disease SMA. The limited number of SMN-positive nuclear-body CBs in motor neurons is a disease’s hallmark and whether it contributes to the pathology is still unclear. We therefore conduct a cell-based assay to screen for small-molecules that localize SMN into CBs and identify flunarizine. We next evaluate *in vivo* its impact on the pathophysiology of the severe Taiwanese SMA mouse model. For the first time, we show a functional link of flunarizine with the CB recruitment of SMN and the splicing snRNA repertoire, and provide several lines of evidence for its beneficial action on spinal cord motor neurons and skeletal muscles in SMA mice. Consistent with earlier hypothesis^15^, the nuclear functions of SMN might be relevant to motor neuron biology and diseases.

Flunarizine treatment restores SMN into CBs of spinal cord motor neurons and increases motor function and lifespan in SMA mice. Beneficial effects on the therapeutically relevant SMA’s hallmarks of SMN-deficient mice are seen with a protection of spinal cord motor neuron cell populations associated to an increase in the number of vGlut1 synapses (Fig. 5C) and a reduction of muscle atrophy in SMA mice (Fig. 7B). Moreover, in line with evidence that muscle defects might precede the loss of mo tor neurons in SMA mice^50,51^, we also observe an increase in the number of muscle fibers in the soleus between 5- and 11-day-old flunarizine-treated SMA mice (Fig. 7C). These results could reveal that the drug might modulate post-natal muscle development that is consistent with observations reported in severe SMA mice and patients^52–54^. Impairments in size, maturation and maintenance of the neuromuscular synapses are also found in SMA mice^48,55^. Flunarizine attenuates those alterations as shown with an increase of more mature neuromuscular synapses in the soleus and of their size in the plantaris and tibialis of mutants (Fig. 8). The differences in response to flunarizine of individual muscle may result from variations in the maturation status, the soleus being the least mature of the three muscles.

Our observations suggest that flunarizine protects and functionally enhances the spinal cord motor neurons in SMA mice, presumably through both cell- and non-cell-autonomous mechanisms. Motor neurons receive different synapses connecting the central and peripheral nervous systems to activate skeletal muscle contraction. The contraction generates feedback signals in sensorimotor circuits as reflected, in part, by vGlut1 synapses on motor neurons. Here, we show that flunarizine diminishes the abnormalities in motor neuron synaptic connectivity of SMA mice. Our results are surprising given the relative small amount of compound used (daily dose of 0,5 mg/kg has a human equivalent dose (HED) of 0,03 mg/kg for a child of 10 kg, while the approved dose in clinics is 0,5 mg/kg). This has some advantages since we do not observe side effects of the drug on the neuromuscular system as indicated with no biological effect on blood vessels in SMA muscles (Fig. S3). Flunarizine also prolongs lifespan in SMN-deficient mice (Fig. 2A). As previously suggested the mortality in SMA mice is probably due to unrelated events induced by SMN deficiency in a wide range of tissues and the long-term survival must therefore involve a systemic rescue^56^.

Other findings indicate that low levels of SMN cause alterations of the splicing snRNA repertoire that can impact directly or indirectly on splicing, mRNA transport and translation^25,26,57^. An elegant study has also demonstrated that mature snRNPs can rescue motor neuron defects in a SMN-knockdown zebrafish model^58^. Consistent with previous results in the SMNΔ7 SMA mouse model^21,22^, we observe here a reduction of the minor spliceosomal snRNAs U12 and U4atac in tissues of the Taiwanese SMA mice that are not increased by flunarizine (Figs 4A and 7D). We show that the molecule rather leads to reduced levels of the major spliceosomal U1, U2, U4 and U5 snRNAs in spinal cord of SMA mice (Figs 4A and 7D), modulating the splicing snRNA repertoire in a tissue-specific manner. We suggest that the competition between the minor and major snRNAs under low levels of SMN would be reduced giving more chance for minor snRNAs to be assembled in spinal cord of flunarizine-treated SMA mice. Although the Taiwanese mouse model is a little more severe^59^ than the SMNΔ7 model (with a shorter lifespan of about 20%), there is no significant difference of snRNA levels in spinal cord of the 5-day-old Taiwanese SMA mice compared to control mice^60^. The disease phenotype starts to be apparent with the 6-day-old SMA mice (Fig. 2B), suggesting that the limiting amount of SMN might provide sufficient snRNAs during early post-natal period.

The molecular mechanisms underlying the snRNA-level changes in SMA are not fully understood^61^. The snRNAs might be degraded by a quality control mechanism sensing defects in the loading of the Sm proteins (the core components of snRNPs) on snRNAs by the SMN complex in the cytoplasm. In the nucleus, other factors localized in CBs also regulate snRNA levels^62–66^. For example, the association of the U4/U6.U5 tri-snRNPs is enhanced with three to four CBs per nucleus^67^ and is reduced in SMA patient cells lacking SMN-positive CBs^24^. Both immature and recycling snRNPs accumulate in CBs thereby maintaining functional snRNPs in the nucleoplasm^68^. Lastly, the snRNA genes across the genome form clusters around CBs, and removal of CBs reduces gene clusters leading to decreased snRNA levels^69^. We suggest that the localization of SMN into CBs upon flunarizine treatment might contribute to the effects on snRNA levels. Moreover, our study may have implications in other neurodegenerative disorders. Perturbations of the splicing snRNA repertoire are also observed in mouse models of adult motor neuron diseases^9^. Loss of motor neurons is also a hallmark of pontocerebellar hypoplasia, an early-onset neurodegenerative syndrome. Recent studies have linked this syndrome to target of early gene 1 member 1 (TOE1) and vaccinia related kinase 1 (VRK1) genes encoding proteins concentrated in CBs and involved in snRNA biogenesis^70,71^. Thus, modulation of nuclear bodies might ameliorate these diseases.

In summary, an early feature of SMA disease is the loss of spinal cord motor neuron synaptic connections that are improved with the flunarizine treatment in SMA mice. Flunarizine is a small molecule classified as a calcium blocker that has been used to treat epilepsies and migraines for years^72^. Mis-splicing of calcium channel subunit genes, alteration of calcium channel clustering at motor neuron axon terminals and intracellular calcium homeostasis have been reported in SMA mouse models^60,73,74^. Other observations on disease manifestations in different neurodegenerative disorders point also to mechanisms regulating calcium homeostasis^75^. Further studies are required to understand the therapeutic potential of this molecule in motor neuron diseases and to identify novel modifier(s) of disease severity. Future *in vivo* experiments will need to address whether flunarizine has effects on splicing in various tissues. Our findings offer groundwork for the development of novel therapeutic approaches directed at modulating the snRNA repertoire and stabilizing synapses against those devastating neurodegenerative disorders.

## Materials and Methods

All methods were carried out in accordance with approved biosafety and radiation guidelines for Université Paris Descartes and adhered to European and French guidelines. All animal experiments were performed following protocols approved by the local animal protection committee (Comité d’Ethique en matière d’Expérimentation Animale Paris Descartes - CEEA 34). All animal procedures adhered to the guidelines of the French Ministry of Education, Research and Innovation. The human skin tissue donation has been authorized by the Assistance Public – Hôpitaux de Paris (Public Assistance – Paris Hospitals) following the guidelines of the French Ministry of Health. All experiments on human cell cultures were performed in accordance with guidelines and regulations of the French Ministry of Education, Research and Innovation.

### Cell culture

Human fibroblasts and HeLa human cervical carcinoma cells were grown in Dulbecco’s modified Eagle’s medium (DMEM)-Glutamax supplemented with 10% fetal bovine serum (FBS), penicillin (100 U/ml), streptomycin (100 mg/ml) and 5% CO_2_. The different skin fibroblast cell cultures were derived from infantile SMA type I, II and III patients with different copy number of SMN2 genes (Table S4 in supplemental material) and informed consents were obtained from patients or their parents (cells were kindly provided either by Pr J Melki, INSERM, France or by MyoBank-Généthon, Evry, France see website: institut-myologie.org/en/recherche-2/myobank-afm/). The SV40-SMA cell line was generated using a primary fibroblast culture derived from a SMA type I patient transfected with a plasmid for the expression of the SV40 large T antigen under G418 selection and previously published^37^.

#### Immunofluorescence analysis

The cells grown on culture-slides were washed with PBS and fixed with 4% formaldehyde (FA) in PBS, permeabilized with 0.5% Triton X-100 and immunostained as previously described^76^, using primary anti-SMN, anti-coilin and anti-TMG antibodies detailed in Supplemental Table S6. The final wash contained 0.1 mg/ml 4, 6-diamidino-2-phenylindole (DAPI; Molecular probes) to stain DNA and ensure that no cytoplasmic staining of mycoplasma is detected.

#### Protein gel electrophoresis and immunoblot analysis

The proteins were resolved on 10% ProSieve 50 polyacrylamide gel (FMC Bioproducts, Rockland, ME) in Tris-Tricine running buffer, transferred to PVDF membranes (Millipore), and immunoblotting experiments were performed as described^2^, with antibodies detailed Supplemental Table S6. After the washing step of a 45-min incubation with HRP-conjugated secondary antibodies, the proteins were visualized using chemiluminescence (Amersham ECL, GE Healthcare).

### Compound preparation

The compounds were supplied from Sigma-Aldrich as three small Lopac libraries, L6912 (ion channel modulator ligand-set, 80 small molecules), L2538 (purines/pyrimidines ligand-set, 64 molecules) and L6662 (serotonergic ligand-set, 80 molecules). They were dissolved in DMSO at a concentration of 2 mg/ml as a stock solution for the culture cell screening, following the supplier’s recommendations. The solutions were further diluted with culture cell medium to obtain 2 μg/ml. For drug exposure, the human fibroblasts were plated in eight-chamber culture-slides (Becton Dickson Laboratory) at a density of 10 000 cells/cm^2^ in DMEM-Glutamax supplemented with dialysed FBS. The concentration of DMSO in each well of the culture-slides was 0.1% and no significant effect was observed.

For *in vivo* studies, flunarizine was dissolved in DMSO at a concentration of 50 mg/ml as a stock solution and diluted in sterile saline solution (0.9% NaCl) to obtain 500 μg/ml and daily injected at a dose of 0.5 mg/kg (1 μl/g).

### RNA preparation and RT-qPCR

Total RNA was extracted using Trizol Reagent (Invitrogen Ambion) according to the manufacturer’s recommendations, followed by a RQ1 RNase-free DNase treatment (Promega) and a cDNA synthesis from 1 μg RNA using miScript II RT kit (Qiagen). Quantitative real-time PCR was performed in triplicate with diluted cDNA (either 1/1000 or 1/10000, for minor or major spliceosomal snRNAs respectively) using SYBR Green ROX Mix (Thermo Scientific) with an Applied Biosystems 7500 fast system. Three independent experiments were carried out for each condition. The snRNA, 5 S and 5.8 S primers have been previously described^22,25^. The normalized expression levels were calculated according to the ΔΔCt method to establish the relative expression ratio between the vehicle-treated control mice and the other groups of mice. The analyses of the SMN2 exon 7, SNAP25 exon 5 and AGRN Z’exon were performed as described^26,39,77^. Additional primers have been designed. All primers are listed in Table S5 in the supplemental material.

### SMA mouse models

Institutional animal care and use committee at Université Paris Descartes has approved protocols in accordance with the national authority (Ministère de l’Enseignement Supérieur, de la Recherche et de l′Innovation, France) guidelines based on European Union Directive 2010/63/EU. The local animal protection committee (Comité d’Ethique en matière d’Expérimentation Animale Paris Descartes - CEEA 34) approved our project and all *in vivo* experiments (including anaesthesia and euthanasia when required) were performed under the reference number 01246.02 and B75-06-07. The criterion for euthanasia was when the mice were no longer able to stand up 20 s after being put on their side^39^. The Taiwanese SMA mice (FVB.Cg-Tg(SMN2)2Hung*Smn1*^tm1Hung^/J strain)^38^ were obtained in collaboration directly from Hung Li’s Laboratory, Institute of Molecular Biology, Academia Sinica, Nankang, Taipei 115, Taiwan. The SMA mice are on FVB/NRj background (Janvier, Le Genest -St-Isle, France) and have been backcrossed for 10 generations as described^42^. The severe Taiwanese SMA model (Smn^ko/ko^; SMN2^tg/0^) and the corresponding control heterozygous (Smn^ko/wt^; SMN2^tg/0^) mice were produced using 2 breeding schemes as previously described^38,59,78^. The average litter size was 8 pups with 12.5% or 50% SMA pups for an average of 31.5% SMA pups per litter. The SMA mice have a mean survival of ≈12 days. Mouse genotyping was performed using DNA extracted from finger biopsies at P6 and primers previously described^38^. Females and males have been equally included in the study. A number was assigned to each animal, and unless stated otherwise, the experiments were performed blinded for genotype, treatment and molecular and cellular analyses.

### Flunarizine administration to animals, analysis of survival, motor function and behaviour

Flunarizine was prepared as mentioned above. The vehicle solution was made of sterile saline solution containing 1% DMSO. We administrated the preparations by intrathecal injections using a Hamilton syringe of 10 μl 1701 RN (#074494, Dutscher SA) added with needle of 33Gx20mm size (#074751 Dutscher SA) at lumbar spinal cord segments 4–5 (L4-5) at a dose of 0.5 mg/kg once daily from birth until the end time point of the experiments, as previously described^39^ and demonstrated in Fig. S4 in supplemental material. This protocol is approved by the local animal welfare and care committee (reference number 01246.02 and B75-06-07). No abnormalities (irritability, convulsion, pain) were observed in mice. The whole litters were treated with the drug or vehicle. The animals were evaluated daily for disease progression. Body weight, motor function in an open field and antigravity hanging tests were recorded before the injection. The first sign of phenotypic difference between SMA mice is in the body weight that was the inclusion/exclusion criteria. The flunarizine- and vehicle-treated SMA mice that would require euthanasia (reduced mobility) were considered in the survival study.

An *antigravity hang test* was performed to estimate the muscle strength of the forelimbs. It consists of measurements of time in seconds the animal hangs to a metal rod suspended in mid-air as previously described^39^. Five measurements for each animal starting at P8 were recorded daily and the highest value was kept for analysis. The hanging time is normalized to the body weight that influences the performance.

An *open-field test* was performed to evaluate the ambulatory behaviour of mice as described^39^. For P8 and P9, the apparatus is a cardboard box (15 × 15 × 7 cm) with the floor of the arena divided into 25 squares of 3 × 3 cm. The 16 squares adjacent to the walls are referred as periphery (dark grey bars) whereas the remaining squares form the center (grey bars) where each animal was placed and allowed to move for 5 min. Starting at P10, the apparatus is a wooden box (28 × 28 × 10 cm) with 16 squares of 7 × 7 cm. The 12 squares adjacent to the walls are referred as periphery whereas the remaining to the center. The measurements were recorded manually.

### Analyses of effects on spinal motor neurons

The SMA mutants and their heterozygote littermates were anesthetized by intraperitoneal injections of pentobarbital (64 mg/kg) and decapitated. For the molecular and cellular analyses, we used vehicle- and flunarizine-treated SMA mice with similar body weight of ≈4 g and 5 g, respectively. Brains and spinal cords were dissected at P10 or P11, snap-frozen (either liquid nitrogen or isopentane) and kept at −80 °C.

#### Localization of SMN in CBs of spinal motor neurons

Fresh cryosections (14 μm) of spinal cords at P11 in isopentane were fixed with 4% PFA in TBS for 20 min, rinsed with TBS-0.1% Tween (TBS-T), incubated in a solution of 1% Triton X-100, 1% Tween in TBS for 30 min at room temperature and with the primary antibodies (Table S6) for 72 h at 4 °C. Following washing at room temperature, sections were incubated with secondary antibodies for 2 h, then with DAPI (nuclei staining) in PBS, washed and mounted in Vectashield (Vectorlabs). Stacks of images were collected at intervals of 0.36–1,0 μm on confocal microscope or on epifluorescence microscope.

#### Central synapses on spinal motor neurons

Spinal cords were dissected at P10 and post-fixed for 16 h, embedded in agarose and sectioned. Sections (50 μm) were incubated in TBS containing 0.2 M glycine for 1 h, washed in TBS-T, blocked with 0.5% Triton X-100, 4% FBS, 1% BSA in TBS-T for 1 h and followed by an incubation for 72 h at 4 °C with primary antibodies (Table S6) diluted in TBS-T containing 0.5% Triton X-100, 0.4% FBS and 0.1% BSA. Sections were washed in TBS-T and incubated for 1 h with secondary antibodies. After incubation with DAPI, sections were washed and mounted.

### Analyses of effects on skeletal muscles

The SMA mutants and their heterozygote littermates were sacrified at P5 and P10 or P11 and three skeletal muscles (soleus, plantaris and tibialis) were dissected, snap-frozen and kept at −80 °C. Muscles were stained as previously described^39^ using the antibodies detailed Table S6.

#### Hematoxylin and eosin staining

Cryosections (10 μm) of mice at P11 were stained with hematoxylin solution for 2 min, eosin solution for 1 min, dehydrated in 3 ethanol bathes (75%, 90% and 100%) of 1 min each and mounted in Eukitt (VWR International) to visualize the morphology.

#### Muscle fiber typology

Cryosections (10 μm) of mice at P11 were fixed with acetone for 20 min, dried and rehydrated (Tween 0.1% in PBS). After 1 h in PBS with 0,1 M glycine, the sections were incubated overnight at 4 °C with antibodies (Table S6) diluted in PBS-T washed and incubated for 1 h with secondary antibody. After DAPI staining and washes, sections were mounted in Vectashield.

#### Neuromuscular junctions

Muscles were dissected at P10, fixed in 4% PFA in PBS for 2 h as whole-mounts and kept in 30% saccharose solution at −80 °C. Muscles were washed in PBS at room temperature and fibers isolated under a benchtop magnifying glass. Fibers were stained by first blocking in 0,1 M glycine in PBS for 1 h and then for 4 h (0.5% Triton X-100, 3% BSA, 5% goat serum in PBS) followed by incubation with primary antibodies (Table S6) for at least 24 h in blocking solution. Fibers were washed for 3 h (0.5% Triton X-100 in PBS) and incubated overnight with secondary antibodies. After washing for 3 h, fibers were incubated for 1 h with Alexa 488 conjugated alpha-bungarotoxine (BGTX, 2 μg/ml, B13422 Molecular Probes) to visualize the NMJs, for 3 min with DAPI (or bis-benzimide), washed for 30 min and flat-mounted in Vectashield.

### Microscope image acquisition and processing

Images were taken using an ORCA Flash 2.8 camera (Hamamatsu Photonics) mounted on an epifluorescence microscope system (AxioObserver Z1, ZEISS). ZEN software was used to acquire and analyse the images. Spinal cord sections were recorded with Z-series containing 0,2-μm sections and deconvolved using AutoQuant X2 software (Media Cybernetics). The number of vGlut1 boutons on motor neuron soma was quantified using the entire Z-series with ImageJ. For the somata size, the plane of the Z-stack with the largest motor neuron area was measured with ImageJ. The size and number of muscle fibers and of NMJs were measured in randomly selected images using ImageJ. For confocal microscopy, sections were images using laser-scanning confocal microscope imaging system (LSM-710, ZEISS) with a 63 × oil-immersion objective. Alexa Fluor 488 (Molecular Probes) and Cy3 (Jackson Laboratories) were used (Table S6).

### Data/Statistical analyses

At least three independent experiments were performed and presented as the mean ± SD or SEM. Statistical analyses were conducted using the non-parametric Kruskal Wallis followed by Dunn’s multiple comparison rank test or two-way ANOVA followed by Tukey’s multiple comparisons test (GraphPad). The values of p < 0.05 were considered statistically significant.

The MyHC data (Fig. 5) were analysed using Stata/IC version 10.1. The distribution for the 4 groups of mice of immuno-labeled or unlabeled fibers for each MyHC isoform was compared using the Kruskal-Wallis rank test (kwallis function). If these distributions are significantly different, an effect of mouse status, treatment, and interaction were secondary tested as an effect of treatment limited to SMA mice. Pairwise correlations using pwcorr function were calculated between the number of immuno-labeled fibers and the body weight of mice. The significant threshold was 5%.

## Electronic supplementary material

Supplementary information
