# Supplementary material for: Small-molecule flunarizine increases SMN protein in nuclear Cajal bodies and motor function in a mouse model of spinal muscular atrophy
Source: Sci Rep. 2018 Feb 1;8:2075. doi: 10.1038/s41598-018-20219-1 (PMC5794986; doi:10.1038/s41598-018-20219-1)
Supplement: Supplementary file 1 — Supplementary information [file 41598_2018_20219_MOESM1_ESM.pdf]

Supplementary Information for

Small-molecule flunarizine increases SMN protein in nuclear Cajal bodies and motor function in a mouse model of spinal muscular atrophy

Delphine Sapaly, Matthieu Dos Santos, Perrine Delers, Gwendoline Quérol, Olivier Biondi, Léo Houdebine, Kevinee Khoobarry, François Girardet, Philippe Burlet, Anne-Sophie Armand, Christophe Chanoine, Jean-François Bureau, Frédéric Charbonnier, Suzie Lefebvre*

*Corresponding author. E-mail : suzie.lefebvre@inserm.fr

This pdf file includes

Tables S1-S6
Figures S1-S5

Supplemental Table 1. Structure of the hits that showed effect on SMN to CBs in fibroblasts derived from a Type I SMA patient

Compound	Name	Source	Structure
G9	Uridine 5'-triphosphate	U4125 Sigma	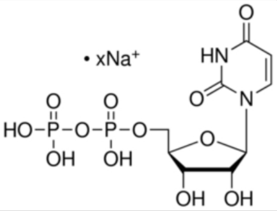
B6	Flunarizine	F8257 Sigma	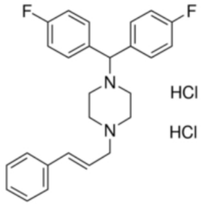
F6	Gabapentine	G154 Sigma	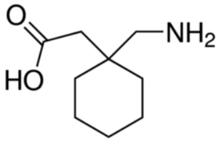
G2	Amiodarone	A8423 Sigma	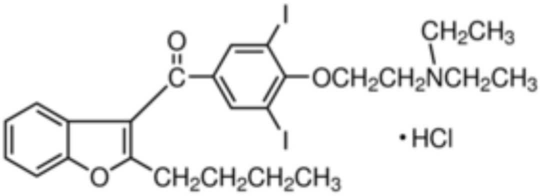
G8	Nitrendipine	N144 Sigma	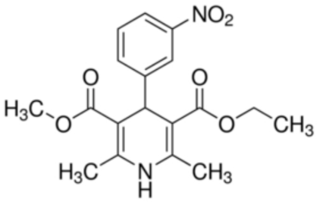
C8	Minoxidil	M4145 Sigma	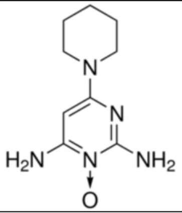
E9	Ro 04-6790	R140 Sigma	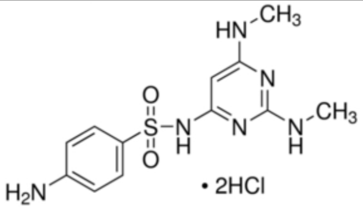

Supplemental Table 2. Activity of positive hits in CB accumulation of SMN protein in fibroblasts derived from a Type I SMA patient

Compound	Concentration of 2 μg/ml (μM)	Max Fold Ind	Dilution at EC50	EC50 (nM)	Fold Ind at EC50
DMSO	-	1±0.3	-	-	-
G9	3.6	3.3±0.7	1:40	90	2.0
**B6**	**4.2**	**4.8±1.5**	**1:53**	**80**	**2.8**
F6	11.6	4.8±1.0	1:55	201	2.8
G2	3.0	2.8±1.0	1:50	60	1.8
G8	5.5	3.2±0.8	1:50	110	2.1
C8	9.6	3.8±1.2	1:9	1060	2.4
E9	5.3	3.4±1.1	1:53	100	2.2

Max Fold Ind corresponds to maximum fold induction.
EC50, concentration required to obtain a response halfway between the baseline and maximum induction.

Supplemental Table 3. Statistical analyses by the Chi-2 test of SMN to CBs in patients affected with SMA presented in figure 1E.

	Type I		Type II		Type III
Compound	Patient GM03813*	Patient 2	Patient 3	Patient 4	Patient 5
G9	3.3	**20.4**	3.5	**11**	0.6
B6	2.9	**27.4**	**6.1**	**11**	**12.4**
F6	**5.4**	**36.9**	3.1	0.8	**5.9**
G2	0.5	**7.2**	0	1.2	2.7
G8	1.9	**32.9**	2.4	0	2.7
C8	2.5	**34.8**	2.0	**5.1**	2.7
E9	4.3	**30.9**	0.7	2.7	2.4

*, from Coriell Cell Repositories, Coriell Institute, USA.

A probability value of 5%, 2%, 1% or 0.1% matches with Chi-2 value of 3.84, 5.41, 6.64 and 10.83, respectively. Significant Chi-2 value ($p < 0,05$) is indicated with bold numbers.

Supplemental Table 4: Copy numbers of SMN1 and SMN2 genes of fibroblasts derived from SMA patients

Cell	Code	SMA type	SMN1 copy number	SMN2 copy number
Patient #1	GM03813 (Coriell)	I	0	3[1]
Patient #2	05400	I	0	3
Patient #3	32829	II	0	4
Patient #4	5-12045 (Généthon)	II	0	3
Patient #5	67457	III	0	1

1. Naryshkin, N.A. et al. SMN2 splicing modifiers improve motor function and longevity in mice with spinal muscular atrophy. *Science* **345**, 688-693 (2014).

Supplemental Table 5 : Primers used for RT-PCR

Primer	Sequence	Source
U1F	GATACCATGATCACGAAGGTGGTT	Ref#22
U1R	CACAAATTATGCAGTCGAGTTTCC	
U2F	TTTGGCTAAGATCAAGTGTAGTATCTGTTC	Ref#22
U2R	AATCCATTTAATATATTGTCCTCGGATAGA	
U4F	GCGCGATTATTGCTAATTGAAA	Ref#22
U4R	AAAAATTGCCAATGCCGACTA	
U5mF	TACTCTGGTTTCTCTTCAGATCGTATAAAT	Ref#22
U5mR	AATTGGTTTAAGACTCAGAGTTGTTCCT	
U6F	GCTTCGGCAGCACATATACTAAAAT	Ref#22
U6R	ACGAATTTGCGTGTCATCCTT	
U11F	GTGCGGAATCGACATCAAGAG	Ref#22
U11R	CGCCGGGACCAACGAT	
U12mF	AACTTATGAGTAAGGAAAATAACGATTCG	Ref#22
U12mR	CCGCTCAAAAATTCTTCTCACA	
U4atacF	TTTCTTGGGGTTGCGCTACTGT	Sref1
U4atacR	AAAGCAGAGCTCTAACCGATGCAG	
U6atacF	AGGTTAGCACTCCCCTTGACAA	Ref #22
U6atacR	TGGCAATGCCTTAACCGTATG	
SMN2ex4-5-F	TGTGTTGTGGTT- TACACTGG	Ref #39
SMN2ex4-5-R	TATT- TCCAGGAGACCTGGAG	
SMN2ex7-8-F	AAAAAGAAGGAAGGTGCTCAC	Ref #39
SMN2ex7-8-R	GCCTCACCACCGTGCTGG	
hSMNint3-F	CGAGATGATAGTTTGCCCTCTTC	Ref #33
hSMNint3-R	TCCCCAACTTTCCACTACAAAAG	
AGRN-31F	TGTCCTGGGGGCTTCTCTGG	Ref#79

AGRN-34R	CAACCTTTCCAATCCACAGCACC	
SNAP25-F	CCCACCACTACCATGGCCGAAGAC	Ref#78
SNAP25-R	CGGAATTCTTAACCACTTCCCAGCATCTT	
TXNIP-3F	GTGTCCCTGGCTCCAAGAAA	herein
TXNIP-3R	GAGAGTCGTCCACATCGTCC	
mMPZ-F1	CTGGTCCAGTGAATGGGTCT	Sref2
mMPZ-R1	ATGACAATGGAGCCATCCTT	
mTTYH3_1-F	GCCTGGTGCGTCATCATCA	herein
mTTYH3_1-R	CGTAGTGAGTAGGTGGCTCG	
mTTYH3_3-F	GAGTGCACATGCCCAGTTTG	herein
mTTYH3_3R	TTTGGCTCTCATGCTGGAGG	
5S-F	CGGCCATACCACCCTGAAC	Ref #22
5S-R	GCGGTCTCCCATCCAAGTAC	
5.8S-F	CGGCTCGTGCGTCGAT	Ref #22
5.8S-R	CCGCAAGTGCGTTCGAA	
mSDHA-F	GGAACACTCCAAAAACAGACCT	Sref3
mSDHA-R	CCACCACTGGGTATTGAGTAGAA	
mRPL13A-F	AGGGGCAGGTTCTGGTATTG	Sref4
mRPL13A-R	TGTTGATGCCTTCACAGCGT	
mPPIA-F	GGCAAATGCTGGACCAAAC	Sref4
mPPIA-R	CATTCCTGGACCCAAAACG	
mHPRT1-F	TCAGTCAACGGGGGACATAAA	PrimerBank ID 7305155a1
mHPRT1-R	GGGGCTGTACTGCTTAACCAG	
mACTB-F	CCTTCTTGGGTATGGAATCCTGT	Sref4
mACTB-R	CACTGTGTTGGCATAGAGGTCTTTAC	

Supplemental references

Sref1. Lotti, F., Imlach, W.L., Saieva, L., Beck, E.S., Hao le, T., Li, D.K., Jiao, W., Mentis, G.Z., Beattie, C.E., McCabe, B.D. & Pellizzoni, L. An SMN-dependent U12 splicing event essential for motor circuit function. *Cell.* **151**, 440-454 (2012).

Sref2. Hunter, G., Aghamaleky Sarvestany, A., Roche, S.L., Symes, R.C. & Gillingwater, T.H. SMN-dependent intrinsic defects in Schwann cells in mouse models of spinal muscular atrophy. *Hum Mol Genet*. **23**, 2235-2250 (2014).

Sref3. Vandesompele, J., De Preter, K., Pattyn, F., Poppe, B., Van Roy, N., De Paepe, A., & Speleman, F. Accurate normalization of real-time quantitative RT-PCR data by geometric averaging of multiple internal control genes. *Genome Biol*. **3**, RESEARCH0034 (2002).

Sref4. Gong, H., Sun, L., Chen, B., Han, Y., Pang, J., Wu, W., Qi, R. & Zhang, T.M. Evaluation of candidate reference genes for RT-qPCR studies in three metabolism related tissues of mice after caloric restriction. *Sci Rep.* **6**, 38513 (2016).

Supplemental Table 6. Antibodies used for immunodetection studies.

Target	Source	Host species	Concentration used		
			tissues	in vitro	immunoblot
SMN	4B3[73]	mouse	-	1:1000	1:5000
SMN	#502 (peptide, CDIWDDTALIKA YDK)✚	rabbit	1:400	-	-
coilin	Santa Cruz (sc-32860)	rabbit	-	1:1000	-
coilin	5P10*	mouse	1:100	-	-
coilin	Sigma (C1862)	mouse	-	1:1000	-
TMG	Calbiochem (NA02)	mouse	-	1:4000	-
ChAT	Millipore (AB144P)	goat	1:400	-	-
V-GLUT1	Millipore (AB5905)	guinea pig	1:8000	-	-
Neurofilament L	Millipore (AB9568)	rabbit	1:400	-	-
SNAP25	Sigma (HPA001830)	rabbit	1:200	-	-
MyHC-I (MYH7)	Sigma (M8421)	mouse	1:1000	-	-
MyHC-II	Santa Cruz (sc-58797, clone MY-32)	mouse	1:20	-	-
MyHC-IIa (MYH2)	DSHB (A4.74)	mouse	1:20	-	-
MyHC Emb (MYH3)	Santa Cruz (sc-53091, clone F1.652)	mouse	1:20	-	-
MyHC neonat (MYH8)	DSHB (N3.36)	mouse	1:20	-	-
Mouse antibody (HRP)	GE Healthcare (NA931V)	sheep	-	-	1:10000
Mouse antibody (Cy3)	Jackson (115-165-146)	goat	1:400	1:400	-
Mouse antibody (Alexa 488)	Invitrogen (A11001)	goat	-	1:400	-
Rabbit antibody (HRP)	GE Healthcare (NA934V)	donkey	-	-	1:10000
Rabbit antibody (Cy3)	Jackson (111-165-144)	goat	1:400	-	-
Rabbit antibody (Alexa 488)	Invitrogen (A11034)	goat	1:400	-	-
Rabbit antibody (Alexa 488)	Invitrogen (A11008)	goat	-	1:400	-
Goat antibody (Cy3)	Jackson (705-166-147)	donkey	1:400	-	-
Guinea pig antibody	Jackson (706-545-148)	donkey	1:400	-	-

✚ validated by ELISA, western blots and immunofluorescence detection with SMA cells.

* generously provided by Professor M Carmo-Fonseca from reference Almeida, F., Saffrich, R., Ansorge, W. & Carmo-Fonseca, M. Microinjection of anti-coilin antibodies affects the structure of coiled bodies. *J Cell Biol*. **142**, 899-912 (1998).

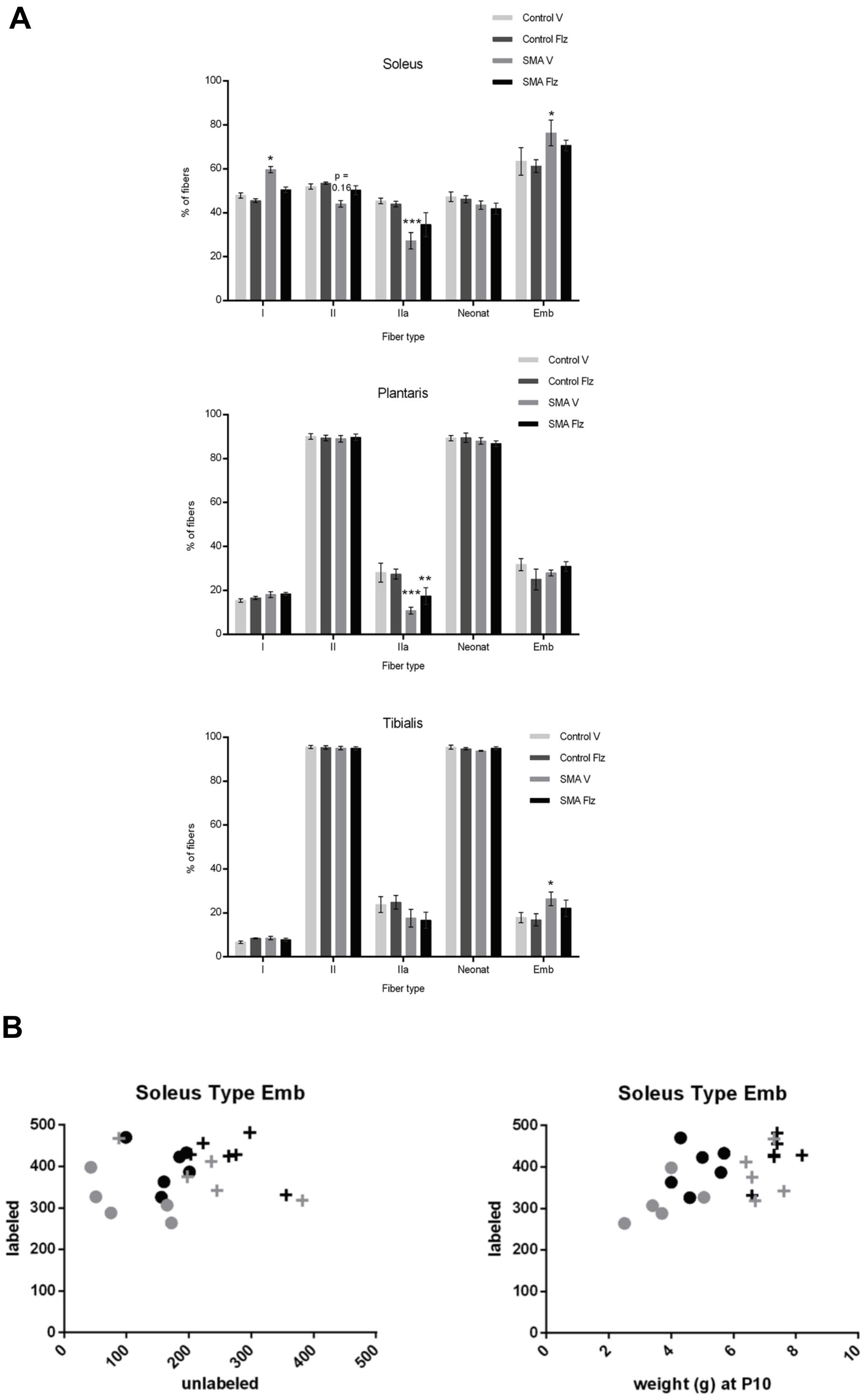

Supplemental Figure 1. Proportion of myofibers in control and SMA mice. A) The different MyHC isoforms are detected by immunofluorescence using specific antibodies. Values are the means ± SEM. (n ≥ 6 mice per group). The Taiwanese SMA mouse model reveals a deficiency in type 2A myofibers as shown previously41. The flunarizine treatment mitigates this defect in the soleus and plantaris of SMA mice. B) The graphs show the number of immunolabeled embryonic fibers (y axis) versus unlabeled fibers or body weight (x axis) for the soleus of 10-days old mice for n≥ 5 mice per experimental group.

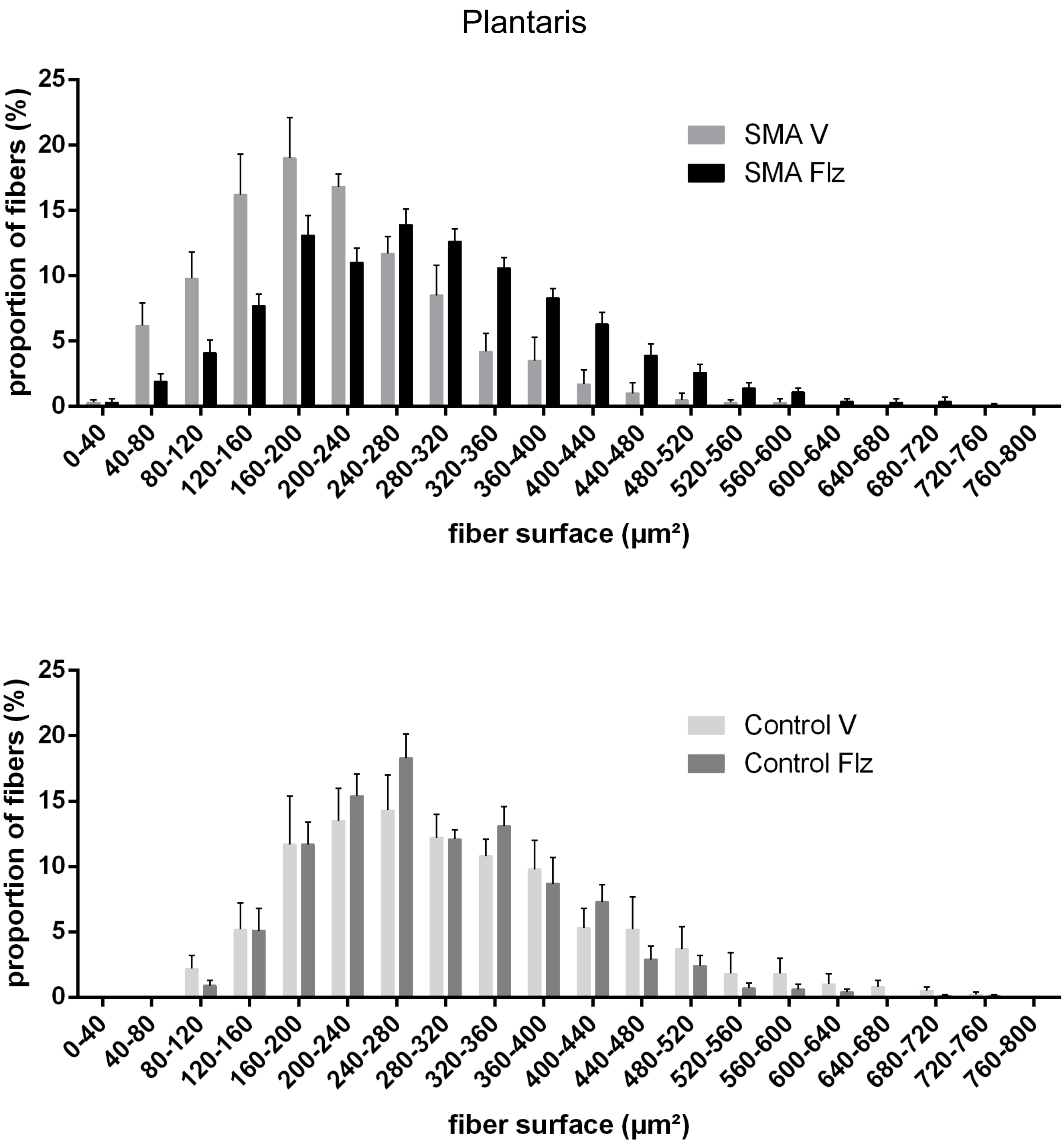

Supplemental Figure 2. Flunarizine completely corrects the muscle atrophy in the plantaris of SMA mice. The distribution of the size fibers in the plantaris of vehicle (V)- and flunarizine (Flz)-treated SMA and control mice are presented (n ≥6 mice per group). Values are the means ± SEM.

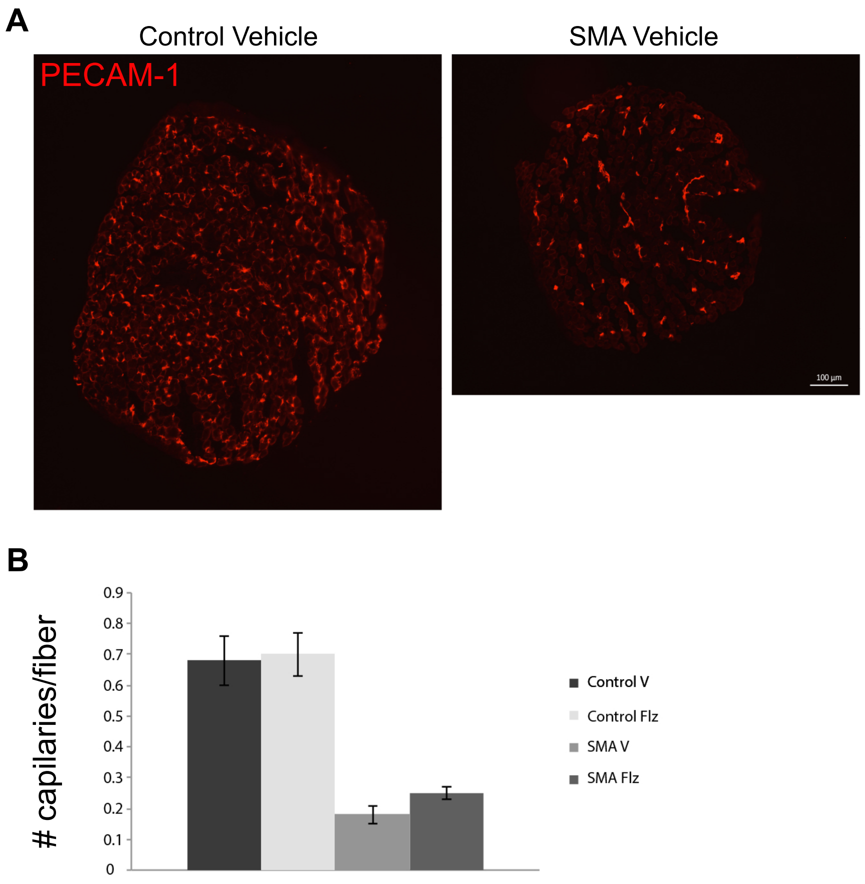

Supplemental Figure 3. The density of capillaries is reduced in muscles from SMA mice. A) Immunofluorescence labeling of cross-sections of the plantaris of vehicle-treated control and SMA mice using a specific antibody against the platelet endothelial cell adhesion molecule (PECAM)-1 protein (1:50, Fisher Scientific sas). B) Frequency histograms depict the number of capillaries per myofibers in the plantaris of 10-day-old mice. A marked reduction of the number of capillaries is observed in SMA mice as previously reported (Somers E. Neuromuscul. Disord., 22, 435–442 (2012)). A very modest increase is obtained with flunarizine in SMA mice.
Values are the means ± SEM. Three mice per experimental group. Chi2 test, p<<<0.001

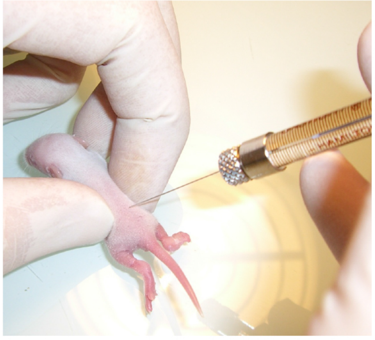

Supplemental Figure 4. Intrathecal injection of mice.

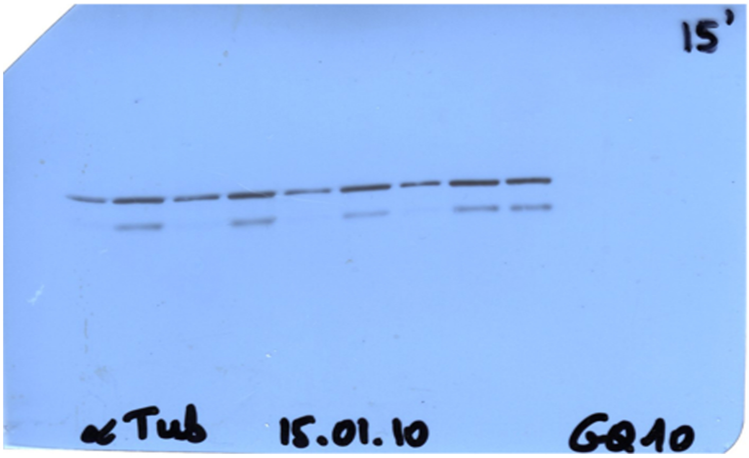

Supplemental Figure 5. Full-length western blot of Figure 1C.
